# Soma influences GSC progeny differentiation via the cell adhesion-mediated steroid-*let-7*-Wingless signaling cascade that regulates chromatin dynamics

**DOI:** 10.1242/bio.201410553

**Published:** 2015-02-06

**Authors:** Annekatrin König, Halyna R. Shcherbata

**Affiliations:** Max Planck Research Group of Gene Expression and Signaling, Max Planck Institute for Biophysical Chemistry, Am Fassberg 11, 37077, Göttingen, Germany

**Keywords:** *Drosophila*, Oogenesis, Germline Stem cell, Ecdysone, miRNA *let-7*, Abrupt, Wingless signaling, Histone modifications, H2Bub1, Differential cell adhesion, Differentiation niche, Cell fate

## Abstract

It is known that signaling from the germline stem cell niche is required to maintain germline stem cell identity in *Drosophila*. However, it is not clear whether the germline stem-cell daughters differentiate by default (because they are physically distant from the niche) or whether additional signaling is necessary to initiate the differentiation program. Previously, we showed that ecdysteroid signaling cell non-autonomously regulates early germline differentiation via its soma-specific co-activator and co-repressor, Taiman and Abrupt. Now, we demonstrate that this regulation is modulated by the miRNA *let-7*, which acts in a positive feedback loop to confer ecdysone signaling robustness via targeting its repressor, the transcription factor Abrupt. This feedback loop adjusts ecdysteroid signaling in response to some stressful alterations in the external and internal conditions, which include temperature stress and aging, but not nutritional deprivation. Upon *let-7* deficit, escort cells fail to properly differentiate: their shape, division, and cell adhesive characteristics are perturbed. These cells have confused cellular identity and form columnar-like rather than squamous epithelium and fail to send protrusions in between differentiating germline cysts, affecting soma-germline communication. Particularly, levels of the homophilic cell adhesion protein Cadherin, which recruits Wg signaling transducer β-catenin, are increased in mutant escort cells and, correspondingly, in the adjacent germline cells. Readjustment of heterotypic (soma-germline) cell adhesion modulates Wg signaling intensity in the germline, which in turn regulates histone modifications that promote expression of the genes necessary to trigger early germline differentiation. Thus, our data first show the intrinsic role for Wg signaling in the germline and support a model where the soma influences the tempo of germline differentiation in response to external conditions.

## INTRODUCTION

In a multicellular organism all cells are united to provide the best response to ever-changing internal and external cues, offering the optimal conditions for organism welfare. This concerted action of different cell types is regulated at different levels, for example, hormones provide systemic signaling for the whole organism. However, cells can communicate more locally within organs or even talk to individual cells from other tissues using a lexicon of different signaling pathways, the majority of which are highly evolutionary conserved. In most cases, signaling molecules or ligands are distributed in the extracellular matrix and are diffused within few cell diameters (e.g. BMP, Hh, Wnt); sometimes, ligands are directly transmitted between the neighboring cells (e.g. Notch signaling). In addition, cells can converse via cell adhesion contacts that adjust tissue maintenance, form and function. All these communication modes are particularly important during embryonic development, but also play an essential role during adulthood for growth, homeostasis and tissue regeneration under certain physiological and pathological conditions. Although a great deal is known about the role of different signaling pathways for development and maintenance of different cells and tissues, it remains challenging to hierarchically connect different levels of cell communication to clearly understand how signals received by one cell type are transmitted to regulate the fate of another cell.

In all organisms, the mature egg production is known to be one of the most highly regulated events; therefore, this process can serve as a great paradigm to study the hierarchical signaling cascade that involves communication between the two cell types of extremely different origin: the germline and the soma. In general, the decision to produce a mature egg is based on the whole organism status and specific tissues, which greatly depend on age, health, nutrition, etc. Currently the knowledge at the molecular level of how germline differentiation is regulated in adults is limited; therefore, it is important to identify the extracellular ligands, membrane receptors and transcription factors involved in the signal transduction pathways that dynamically guide oocyte maturation to reach a consensus between changing internal states and external environments.

In mammals, oocytes undergo an extensive maturation process that is carefully controlled and recent progress highlighted that, besides paracrine signals, cell to cell interactions with surrounding somatic cells play important roles in oocyte differentiation (Murray et al., 2010; Li and Albertini, 2013). Somatic epithelial granulosa cells that surround the developing oocytes are required to control oocyte meiotic arrest and growth (Von Stetina and Orr-Weaver, 2011). Following puberty, the luteinizing hormone acts on granulosa cells, stimulates the activation of EGFR and subsequently MAPK kinase signaling, which then causes reversal of the inhibitory signals that are sent to the oocyte. In this manner, systemic signals are integrated in somatic granulosa cells to regulate oocyte differentiation. In addition, multiple components of the Wnt/Wingless (Wg) signaling pathway are expressed in the adult ovary. During adulthood, the monthly fluctuations in Wnt/β-catenin signaling are balanced by sex hormones in the endometrium (soma) to manage estrogen-induced proliferation and progesterone-induced oocyte differentiation (germline). Abnormal Wnt/β-catenin signaling strength in gonads causes reproduction defects and cancer (van der Horst et al., 2012). Wnt/Wg pathway is highly evolutionary conserved and is considered as one of the most important developmental pathways (Clevers and Nusse, 2012). A key Wnt/Wg signaling mediator is the nuclear transcription factor β-catenin, which also is a binding partner of the major component of adherens junctions, E-Cadherin. β-catenin levels not only affect the cell adhesiveness, but also the expression profile of multiple genes, as Wnt/Wg signaling cell autonomously regulates gene expression via interaction with chromatin modifying complexes (Liu et al., 2008; Parker et al., 2008; Saito-Diaz et al., 2013). Slight variations in β-catenin amounts and/or its cellular localization have a profound effect on cell status. While functional studies indicate that Wnt/Wg signaling has a role in several aspects of ovarian function including folliculogenesis and steroidogenesis (Boyer et al., 2010; Usongo et al., 2012), a number of questions regarding its functions in the germline remain open.

*Drosophila* ovary provides an excellent system to study at the molecular level how germline differentiation is adjusted in response to dynamic internal and external conditions. *Drosophila* oogenesis depends on the presence of adult germline stem cells (GSCs) that continuously divide. Mostly, stem cells divide asymmetrically when a mother cell gives rise to two daughter cells with different fates – another stem cell and a differentiating progeny (Gönczy, 2008). Alternatively, two daughters may be identical at birth and their fate is established later on, for instance through signaling from neighboring cells. *Drosophila* GSCs are an example of the latter, since the stem cell fate of the newborn germline cell depends on the signaling provided by the surrounding soma called the GSC niche (Losick et al., 2011). It is known that exit from the niche abolishes stemness, but it is not clear what combination of signals promotes germline differentiation. Physiologically, it seems likely that signaling that coordinates the GSC progeny differentiation and egg maturation efficiency with the whole organism needs and conditions exists. While a lot is known about GSC maintenance and division regulation upon different conditions, the questions what makes stem cell daughter to differentiate and whether the differentiation process *per se* can be regulated in response to physiological state of the whole organism have not been analyzed in depth.

Our previous data provide evidence that ecdysone signaling acts in the soma: (1) during pre-adult stages, to cell autonomously regulate the size of the GSC niche, and (2) during adulthood, to cell non-autonomously regulate the germline differentiation speed via the somatic cells of the differentiation niche (König et al., 2011). In this study we aimed to understand how information is exchanged between the soma and germline, specifically how changes in the somatic cells in the adult ovary are communicated to the germline and regulate germline differentiation. We found that ecdysteroids regulate cellular identity of escort cells (ECs), comprising the differentiation niche, which is juxtaposed to the GSC niche to coordinate the speed of the early GSC progeny differentiation. Depending on the ecdysone signaling strength, cell shape, proliferative ability and, most importantly, adhesive characteristics of ECs are modified, together resulting in squamous to cuboidal-like epithelium transformation. The epithelial state depends on the function of the BTB transcription factor, Abrupt (Ab), subcellular localization of which is dose-dependent and is regulated by ecdysone signaling. This regulation in addition is fine-tuned by the steroid-induced miRNA *let-7*, which acts in a feedback loop to reinforce ecdysone signaling via Ab downregulation, since Ab also is a repressor of ecdysone signaling. Importantly, alterations in the EC adhesiveness influence the presentation of the cell adhesion proteins in the germline cells, because ovarian soma and the germline are connected via homophilic cell adhesion mediated by cadherins. As cadherin levels must match on the membranes of both cell types, somatic and germline, an increase or decrease of adhesion molecule amounts is immediately communicated to the other cell type via direct cell-cell contacts. Cadherins also have an ability to bind signaling molecules, for example DE-Cadherin (DE-Cad) binds Armadillo (Arm, *Drosophila* β-catenin), which in turn modulates the Wg signaling strength. Thus, ecdysone signaling in the soma influences Wg signaling in the germline via direct cell-cell contacts. The role for the Wg pathway in the *Drosophila* germline has not been reported previously, our data show that the Wg signaling intensity positively affects the early germline differentiation speed. Wg-mediated regulation of the GSC progeny differentiation occurs at the chromatin modification level that controls the initial steps of the GSC daughter decision to enter the differentiation program. Upon decreased Wg signaling, the GSC progeny is caught in the “pre-CB” state: it is not a stem cell anymore, since it cannot perceive signaling from the stem cell niche; however, it is not a differentiating CB yet, since its chromatin remains in the “stem-cell-like” state and is not properly modified to allow the expression of genes necessary for differentiation (e.g. *bam*). In particular, histone H2B monoubiquitination (H2Bub1) is affected upon ecdysone and Wg deficit, postponing *bam* expression and the pre-CB entrance into the differentiation program. In summary, we show that systemic steroid hormone signaling fine-tunes the tempo of GSC progeny differentiation in response to environmental fluctuations. It acts in the somatic differentiation niche to cell non-autonomously, via adjustment of cell adhesion complexes, manage the Wg signaling strength in the germline cells, which modulates their chromatin state, favoring differentiation.

## MATERIALS AND METHODS

### Fly strains and genetics

Flies were raised on standard cornmeal-yeast-agar-medium at 25°C and fattened on wet yeast paste one day before dissection unless otherwise stated. *w^1118^* and *OregonR* were used for controls. The two knockout strains *let-7-C^GK1^/CyO* and *let-7-C^KO1/^CyO* lack the whole *let-7-C*, in addition *let-7-C^GK1^/CyO* contains the transcriptional activator *Gal4* under the control of the *let-7-C* promoter (gift from Nicholas Sokol). Flies with a transgene rescuing the whole *let-7-C (let-7-C; let-7-C^GK1^/let-7-C^KO1^)* were referred to as “Rescue” (Sokol et al., 2008). The *let-7-C^Δlet-7^* construct restores expression of all *let-7-C* members except for *let-7*; *let-7-C^GK1^*/*let-7-C^KO1^*; *let-7-C^Δlet-7^* flies, therefore, were, abbreviated as *Δlet-7* (Sokol et al., 2008). *FRT 40A let-7 miR-125/CyO* flies (Caygill and Johnston, 2008) were used for clonal analysis. The following additional fly stocks were used: *ab^1^*, *ab^k02807^*, *w^1118^; ab^1D^/CyO*, *y^1^ w^67c23^; bam^EY04821^*, *y^1^ w^67c23^; bam^EY03755^*, *w^1118^; UASab.B*, *ecd^1ts^*, *EcR^Q50st^*, *FRT 101 arm^2^/FM7a*, *FRT 101 arm^3^/FM7a*, *usp^4^/FM7a*, *hsbam/TM6*, *hsEcR.B1* (BDSC); *UAStai^RNAi^*, *UASab^RNAi^* (VDRC), *bamGFP* (gift from Dennis McKearin), *FRT 101 sgg^D127^/FM7*, *UASp arm* (gift from Andreas Wodarz), *FRT 2A Bre1^P1549^* (gift from Sarah Bray), *FRT 19A usp^4^/FM7* (*usp^4^* allele from BDSC, recombined in this study), *UAS Cad ^(109004)^* (DGRC). The *VALIUM20* lines in which dsRNA expression system was constructed to work in both, the soma and germline: *UASarm^RNAi^* ^(35004)^, *UASfz^RNAi^* ^(34321)^, *UASsgg^RNAi^* ^(38293)^, *UASpan^RNAi^* ^(40848)^ and *UASBre1^RNAi^* ^(35443)^, *UASRtf1^RNAi^* ^(36586)^ (BDSC).

### Perturbation of ecdysone signaling, the Wg pathway or H2B monoubiquitination

The *ecd^1ts^* temperature-sensitive mutation is known to reduce ecdysone levels at the non-permissive temperature. Fly stocks were kept at the permissive temperature (18°C) and 2- to 4 day old adults were shifted to the restrictive temperature (29°C) for 4 days in order to repress ecdysone synthesis. As control, *OregonR* flies were kept at 29°C for the same time. To disrupt ecdysone signaling specifically in the somatic cells of the germarium, *UASab.B*, *UAS let-7* or *UAStai^RNAi^* were expressed using the soma-specific drivers *bab1Gal4*, *ptcGal4* or *let-7-C^GK1^* (contains *Gal4*). In addition, to disrupt ecdysone signaling during adulthood only, the *tubGal80^ts^* system was used where the flies were raised at 18°C and switched to 29°C for 3–5 days. Interaction of ecdysone signaling pathway and Bam was analyzed by heat-shocking *hsEcR.B1/+*, *hsbam/+* and *hsEcR.B1/+*; *hsbam/+* flies for 1 hour, 2 days in a row in a 37°C water bath; not heat-shocked flies of the same genotypes were used as *controls*.

To alter the strength of Wg signaling in the germline, *UASarm^RNAi^*, *UASfz^RNAi^*, *UASarm* or *UASpan^RNAi^* flies were crossed to the germline specific driver *nosGal4* (*NGT40/+; nanosGAL4/+*). To analyze the interaction between ecdysone signaling components and Cad or Arm, dominant-negative mutations of *EcR* and *usp* were used: *w^1118^; hs-Gal4-EcR.LBD/+* or *w^1118^; hs-Gal4-usp.LBD/+* flies were crossed to *shg^E17B^/SM6b*, or *FRT 101 arm^2^/FM7A*. 1–3 day old adult progeny were heat-shocked in empty vials for 60 min per day, 4 days in a row. To analyze the interaction between ecdysone signaling components and Bre1, *EcR^Q50st^/+;FRT 2A Bre1^P1549^/+* flies were analyzed.

For perturbing H2B monoubiquitination in the germline, *Bre1^RNAi^* or *Rtf1^RNAi^* were crossed to germline specific driver *nosGal4* (*NGT40/+; nanosGAL4/+*).

### Clonal analysis

Germline and somatic cell clones were induced as described previously (Shcherbata et al., 2004; Shcherbata et al., 2007) using the *hsFlp/FRT* system for mitotic recombination. *let-7* mutant clones in CpCs and ECs were obtained via crossing *FRT 40A let-7 miR-125/CyO; let-7-C^Δlet-7^* to *FRT 40A Ubi-GFP/CyO; bab1Gal4:UASFlp/TM2* flies (gift from Acaimo González-Reyes). Alternatively, the MARCM system was used and *FRT 40A let-7 miR-125/CyO; let-7-C^Δlet-7^* flies were crossed to *hsFlp*, *FRT 40A tubGal80^ts^/CyO; tubGal4/TM6B*. To induce *usp^4^* mutant clones, *FRT 19A usp^4^/FM7* flies were crossed to *w*, *FRT 19A tubGal80*, *hsFLP; UASnucLacZ*, *UAS CD8GFP; tubPGal4/TM6B* flies (gift from Frank Hirth). Third instar larvae were heat-shocked for 2 hours, 2 days in a row in a 37°C water bath. To induce adult germline clones, *FRT 40A let-7 miR-125/CyO; let-7-C^Δlet-7^* males were crossed to *hsFlp; FRT 40A GFP/CyO* females.

To obtain adult *Bre1* germline clones, *FRT 2A Bre1^P1549^/TM3* flies were crossed to *hsFlp; FRT 2A GFP/TM3*. For inducing *arm^2^*, *arm^3^* and *sgg ^D127^* mutant clones *FRT 101 arm^2^/FM7a*, *FRT 101 arm^3^/FM7a* or *FRT 101 sgg^D127^/FM7* flies were crossed to *FRT 101 GFP; hsFlp/CyO*. 2–4 day old adult F1 females were heat-shocked in empty vials for 60 min, 2 days in a row in a 37°C water bath.

The GSC loss per day is determined by division of the percentage of clonal germaria with lost GSCs by the elapsed time after clonal induction (5 days). For *let-7* mutant clones, females were analyzed 7 and 14 days after heat-shock, for *Bre1^P1549^*, *arm^2^*, *arm^3^* and *sgg ^D127^* mutant clones-5 days after heat-shock. Parental *FRT 40A*, parental *FRT 2A* and parental *FRT 101* flies were used as *controls*. Mutant clones were identified by the absence of GFP; or by the presence of GFP (MARCM).

### Expression patterns of *let-7* and Ab at different conditions

*In situ* hybridization was performed as described previously (Kucherenko et al., 2012). To analyze *let-7* levels at different conditions, germaria of *OregonR* flies were dissected and analyzed using RT-qPCR: young (1–3 days) and old (21 days) flies were compared; flies were kept on rich food or poor food (sugarfree) for 2 days; or were kept at 18°C or 29°C for 2 days. In order to measure the levels of Ab, *OregonR* and *ecd^1ts^* animals were kept at 18°C or 29°C for 4 days and germaria were analyzed using RT-qPCR; *OregonR* animals were heat-shocked for 1 h at 37°C and immediately dissected for immunostaining.

### RT-qPCR

Ovaries were dissected to perform quantitative reverse transcription (RT-qPCR). Eggs and follicles of later stages were removed and only ovary tips containing germaria were used for analysis. RNA was extracted using Trizol according to the manufacturers instructions. For *let-7* RT-qPCR, reverse transcription and qPCR were performed following the manufacturers protocol using TaqMan® MicroRNA assay for *let-7* and for *S2* as endogenous control. For analysis of *RpL32* levels as endogenous control and *esg*, *Imp*, *upd*, *ab*, *arm*, *sgg*, *pan* and *fz* cDNA was generated using the cDNA Reverse Transcription kit (Applied Biosystems) according to the manufacturers instructions. qPCR was performed using the fast SYBR® Green Master Mix (Applied Biosystems). A Step One Plus 96 well system (Applied Biosystems) was used for all analysis, all reactions were run in triplicates with appropriate blanks. The reactions were incubated at 95°C for 20 sec (RpL32) or 10 min (TaqMan® MicroRNA) followed by 40 cycles of 95°C for 3 sec (RpL32) or 15 sec (TaqMan® MicroRNA) and 60°C for 30 sec (RpL32) or 60 sec (TaqMan® MicroRNA). Primers were used as follows: *RpL32* forward – 5′-AAGATGACCATCCGCCCAGC-3′, *RpL32* reverse – 5′-GTCGATACCCTTGGGCTTGC-3′, *esg* forward – 5′-CGCCCATGAGATCTGAAATC-3′, *esg* reverse – 5′-GGTCTTGTCACAATCCTTGC-3′, *Imp* forward – 5′-GGTGGGCCGTATCATTGG-3′, *Imp* reverse – 5′-TCACGCGCTGCAATTCC-3′, *upd* forward – 5′-TTCTGGCTCCTCTGCTGCTTCT-3′, *upd* reverse – 5′-TACCGCAGCCTAAACAGTAGC-3′, *ab* forward – 5′-AGCACCCGATAGTCATCCTG-3′, *ab* reverse – 5′-GGCCTTGGAATAGGGATAGC-3′, *arm* forward – 5′-CCACTGGGCTGCTGATCT-3′, *arm* reverse – 5′-ATGCTTGGACCAGAAGAAGC-3′, *sgg* forward – 5′-ATCAACTTGGTGTCCCTGCT-3′, *sgg* reverse – 5′-GCACTAGGCTGGGCTGTATT-3′, *pan* forward – 5′-AGCGCAGGAACTTTCCATAA-3′, *pan* reverse – 5′-TTGATGTGTGCTTTGCTTCC-3′, *fz* – 5′-GCTGCTTGTTTACGGTGCT-3′, *fz* reverse – 5′-CTGGGTGATGGTGGACAT-3′. The ΔC_T_ value was determined by subtracting the C_T_ value of the endogenous control from the experimental C_T_ value. ΔΔC_T_ was calculated by subtracting the ΔC_T_ of the control sample from the respective ΔC_T_ value. The relative RNA levels were calculated as 2^−ΔΔCT^.

### Analysis of mitotic divisions in ECs

In order to determine whether ECs are mitotically active upon perturbed ecdysone signaling, PH3 antibody was used to detect ECs in M phase and EdU assay in S phase.

### Immunofluorescence and antibodies

Ovaries were fixed in 5% formaldehyde (Polysciences, Inc.) for 10 min and the staining procedure was performed as described (König and Shcherbata, 2013). For H2Bub1 staining: following fixation, ovaries were washed three times for 15 min and ovarioles were separated using needles. After permeabilization (performed in PBS with 2% Triton X) ovaries were incubated in 2N HCl for 30 min at 37°C. The following primary antibodies were used: mouse monoclonal anti-Adducin (1:50), anti-LaminC (1:50), anti-Arm (1:50), anti-Engrailed; (1:50) (Developmental Studies Hybridoma Bank); rat monoclonal anti-DE-Cad (1:50, Developmental Studies Hybridoma Bank); mouse anti-MAP Kinase (1:500, Sigma); mouse anti-H2Bub1 (1:500, Millipore); rabbit anti-pMad (1:5000, D. Vasiliauskas, S. Morton, T. Jessell and E. Laufer); rabbit anti-Abrupt (1:1000 S. Crews); rabbit anti-Vasa (1:5000, R. Pflanz); rabbit anti-β-Gal and rabbit anti-PH3 (1:3000, Upstate Biotechnology); rabbit anti-H3K4me3 (1:1000, Abcam), rabbit anti-H4K20me3 (1:1000), anti-H3K36me2 (1:1000), anti-H3K9me2 (1:1000), anti-H4K20me1 (1:1000), H4 hyperacetylation (1:1000), anti-H3K27me3 (1:1000) (Upstate Biotechnology) and guinea pig anti-Tj (1:3000, D. Godt). Secondary antibodies were used: anti-GFP-directly conjugated with AF488 (1:3000, Invitrogen), Alexa 488, 568, or 633 goat anti-mouse, anti-rabbit (1:500, Molecular Probes) and goat anti-rat Cy5 (1:250, Jackson Immunoresearch). The Click-iT® EdU Cell Proliferation Assay (Invitrogen) was used according to the manufacturers instructions. Images were obtained with a confocal laser-scanning microscope (Leica SPE5) and processed with Adobe Photoshop.

### Analysis and statistics

To determine the number of CpCs, LamC positive cells at the tip of the germarium were counted. Germline cells that were touching CpCs were counted as GSCs and pMad staining was used to prove stem cell identity. To determine the intensity of pMad levels, the gray value of pMad positive cells was measured using Zen Software. The gray value of the background was determined and subtracted from the GSC pMad levels for normalization. GSC maintenance was determined by comparing the percentage of germaria with clonal GSCs between two different time points after clonal induction. Cells that had a single spherical spectrosome but were not in contact with the niche: pre-CBs/pro-CBs/CBs were counted separately and GSCs and pre-CBs/pro-CBs/CBs were added together to calculate the number of spectrosome-containing GCs (SpGCs). In addition, the number of fusomes (indicating the number of differentiating cysts) until region 2b, where follicle cells start cyst encapsulation, was counted. To describe the differentiation efficiency in a given germarium the number of cysts was divided by the number of single spectrosome containing cells (ratio = cysts/SpSCs). In addition, germaria containing clonal mutant germline cysts were analyzed: based on the fusome morphology, the number of cells in a cyst and the location in the germarium, it was compared whether clonal germline cysts were of the same stage of differentiation as their non-clonal neighbors. MAP Kinase, Tj, Cad and Arm staining was used to analyze EC morphology. At least three independent biological replicates were done. The two tailed Student's t-test or two-way tables and *X^2^* test were used to determine the statistical significance.

## RESULTS

### Ecdysone signaling deficiency delays GSC progeny at pre-CB stage by preventing histone modification (H2Bub1) that triggers differentiation

**Fig. 1.**
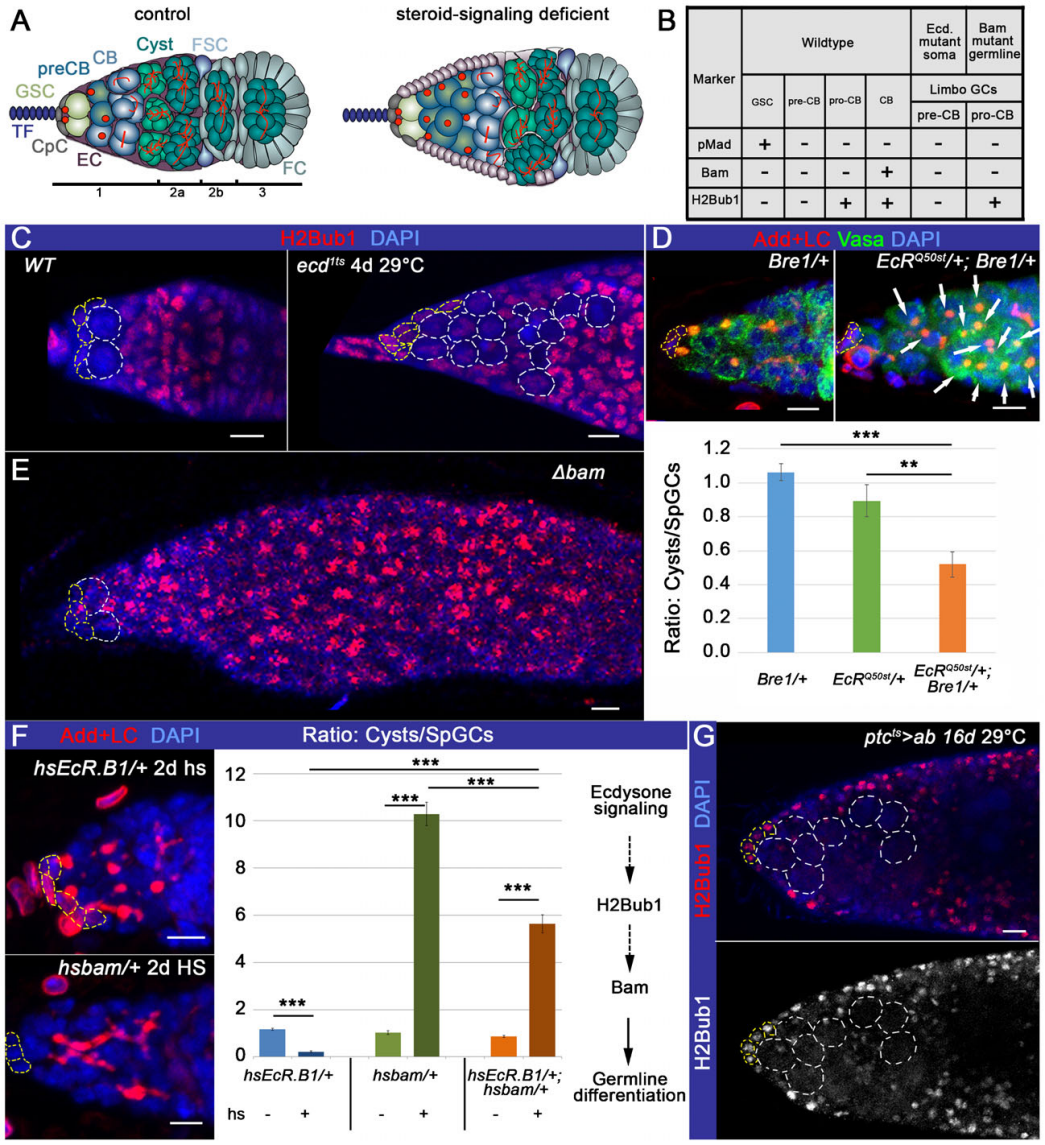
Ecdysone signaling cell non-autonomously regulates GSC progeny chromatin dynamics. (A) Control and ecdysone signaling mutant germaria are compared. In controls, spectrosome-containing germline cells (SpGCs = GSCs+pre-CBs+pro-CBs+CBs) and fusome-containing differentiating cysts are proportionally distributed and ECs form squamous epithelium. In the ecdysone-deficient germarium, supernumerary SpGCs and columnar epithelium-like ECs appear. (B) Table with germline cell markers in wildtype and ecdysone signaling or *bam* mutant germaria. (C) The limbo-GCs in ecdysone signaling mutants (pre-CBs) do not show H2Bub1 staining (*OregonR* and *ecd^1ts^* 4 days at 29°C). (D) Bre1 is required for H2Bub1 modification and, similarly to steroid signaling, affects the efficiency of early germline differentiation (please see supplementary material Fig. S2). *Bre1* genetically interacts with *EcR*, resulting in the decreased differentiation index (Ratio: Cysts/SpGCs; *Bre1^P1549^/+*, *EcR^Q50st^/+*, *EcRQ^50st^/+; Bre1^P1549^/+*, supplementary material Table S1). (E) The ***Δ****bam (bam^EY04821^/bam^EY03755^)* supernumerary SpGCs (pro-CBs) are positive for H2Bub1. (F) Perturbation of ecdysone signaling via *EcR* overexpression (*hsEcR.B1/+*, 2×1 h heat shock at 37°C) leads to a differentiation delay in the GSC progeny, and forced expression of *bam* (*hsbam/+*, 2×1 h heat shock at 37°C) causes GSC loss by differentiation. Overexpression of both proteins (*hsEcR.B1/+*; *hsbam/+*, 2×1 h heat shock at 37°C) overcomes the differentiation delay and leads to the increased differentiation ratio (Ratio: Cysts/SpGCs, supplementary material Table S1). Ecdysone signaling temporally acts upstream of the chromatin modification H2Bub1 and the germline differentiation factor Bam. (G) Similarly, soma-specific ecdysone signaling perturbation (*ptc^ts^>ab: ptcGal4/+*; *tubGal80^ts^/UASab*, 16 days at 29°C) leads to the appearance of supernumerary pre-CBs negative for H2Bub1 and delayed differentiation (supplementary material Table S2). Germaria are stained with H2Bub1 (red, C,E,G), Lamin C (LC red, D,F) to visualize TFs and CpCs and Adducin (Add red, D,F) to mark spectrosomes and fusomes. Nuclei are stained with DAPI (blue, C–G). CpCs are outlined in yellow (C–G), SpGCs are outlined in white (C,G) or indicated with arrows (D). p-values were calculated using the two tailed Student's t-test and error bars represent S.E.M. *p<0.05, **p<0.005, ***p<0.0005. Scale bars, 5 µm.

In *Drosophila*, the oogenesis depends on the presence of GSCs that reside in their stem cell niche, which is located at the apex of the ovariole in the structure called the germarium (Fig. 1A). The GSC niche consists of specialized somatic cells, namely terminal filaments, cap cells and escort cells (TFs, CpCs and ECs, Fig. 1A). These somatic cells make physical contacts with the GSCs via tight, adherens, and gap junctions, the basement membrane and extracellular matrix proteins that often regulate transcription to ensure the stem cell fate maintenance (Song et al., 2002; Gilboa et al., 2003; González-Reyes, 2003; Ward et al., 2006; Hayashi et al., 2009). TGF-β signaling is clearly one of the most important signaling pathways controlling the ovarian GSC population (Chen and McKearin, 2003; Song et al., 2004); it is activated in GSCs by the ligands Dpp and Gbb, which are sent from the niche. As a consequence of this activation, the differentiation factor Bag-of-marbles (Bam) is excluded from the GSCs. Bam is a master differentiation factor that is both necessary and sufficient to induce differentiation in the germline and thus is only expressed in differentiating germline cysts or cystoblasts (CBs) (McKearin and Ohlstein, 1995). In the germarium, there are at least three categories of germline cells (GCs) based on their differentiation state: the GSC maintains undifferentiated stem cell characteristics, the pre-CB lingers in a transient state between stemness and differentiation, and the differentiating CB commits to the egg production program. These germline cell types are proportionally represented in the normal germarium: two to three GSCs, followed by one to three pre-CBs/CBs and four to five differentiating cysts typically are situated in the region 1–2A in the wild type germarium (Fig. 1A). Each germline cell type can be identified using different markers, among which are components of niche-derived TGF-β signaling. Stem cells express the phosphorylated TGF-β transcription factor Mad (pMad), which suppresses the differentiation factor *bam* expression, which is present in the differentiating cysts. Transient GSC progeny or pre-CBs express neither of these markers. In addition, all single cell germline types (GSCs, pre-CBs and CBs) have spherical fusomes (also called spectrosomes), while multicellular cysts are marked with elongated (2- and 4-cell cysts) and branched (8- and 10-cell cysts) fusomes (Lin et al., 1994; McKearin, 1997). Upon ecdysone signaling deficit, the differentiation index measured by the ratio between the numbers of fusome-containing (cysts) to spectrosome-containing germline cells (SpGCs) that do not express either stem cell or differentiation factors is significantly decreased (König et al., 2011). We named these supernumerary SpGCs as “limbo-GCs” due to their delayed in differentiation status (Fig. 1B).

The GSC ability to self-renew and differentiate is often regulated at the level of chromatin structure (Xi and Xie, 2005; Maines et al., 2007; Buszczak et al., 2009; Wang et al., 2011; Yan et al., 2014). Therefore, we analyzed several histone modifications that would be differentially displayed in GSCs *vs* differentiating cysts (supplementary material Fig. S1). Our analysis shows that the majority of histone modifications are present in both, the germline and soma. H3K20me3, H3K4me3, H3K36me2 and H3K27me3 are equally distributed among all somatic cell types in the germarium, while H3K9me2, H4K20me1 and H3K27me3 show differential expression in the CpCs, ECs and FCs. In the germline, 16-cell cysts have higher levels of H3K4me3, H3K36me2 and H4K20me1 than all other differentiating GCs. Among analyzed histone modifications, only monoubiquitination of the histone H2B (H2Bub1) was present in the differentiating germline, but not in stem cells (supplementary material Fig. S1; Fig. 1C). Previously, we have demonstrated that H2Bub1 modification is one of the events that precede entering the differentiation program (Karpiuk et al., 2012). H2Bub1 resolves the bivalency state of numerous genes, which are essential for stem cell progeny differentiation in multiple systems (Johnsen, 2012). Interestingly, the limbo-GCs in *ecd^1ts^* mutants are devoid of this histone modification and rather exhibit the stem cell-like chromatin state (Fig. 1C). To prove that this process is relevant to germline differentiation, we analyzed mutants for *Drosophila* E3 ubiquitin ligase dBre1 required for the histone H2B monoubiquitination (Bray et al., 2005; Mohan et al., 2010). dBre1 was shown to be important intrinsically for GSC maintenance and extrinsically for the germline differentiation (Xuan et al., 2013). Since the differentiating cells in the germarium showed H2Bub1, we considered that this histone modification could be also required intrinsically for efficient germline differentiation. Therefore, we induced *dBre1* loss-of-function clones and found that *dBre1* deficient GSCs that were not lost produced 1.8 times less progeny than control clonal GSCs (supplementary material Fig. S2B,C). Similar delay was observed when *dBre1* or *dRtf1*, the subunit of the Paf1 complex, which we found to be required for proper monoubiquitination of histone H2B in the germline (supplementary material Fig. S2D), were downregulated specifically in the germline (supplementary material Fig. S2E,F). Together, these findings suggest that histone H2B monoubiquitination via Bre1 or Rtf1 is important for successful early germline differentiation. Additionally, we found a genetic interaction between ecdysone signaling and *dBre1* mutants, showing that these pathways are functionally related (Fig. 1D).

Interestingly, H2Bub1 spatial and quantitative expression pattern paralleled with Bam protein levels, implying a correlation between H2Bub1 histone modification and the differentiation factor *bam* expression (supplementary material Fig. S2A). Absence of Bam, similarly to steroid deficit, makes GSC progeny unable to differentiate, and *bam* mutant germline cells also lack all of the before-mentioned markers (McKearin and Ohlstein, 1995; Song et al., 2004). Despite this similarity in TGF-β component expression patterns, there is a critical difference between the limbo-GCs emerging due to *bam-* and ecdysone signaling deficit. While *bam* mutant germline cells have a differentiation block, ecdysone deficit merely causes a differentiation delay. To understand the rationale of this dissimilarity, we tested the H2Bub1 pattern in *bam* and *ecd* mutants and found that *ecd^1ts^* limbo-GCs do not contain H2Bub1, while *bam* limbo-GCs have this histone modification present, just like all other differentiating cells (Fig. 1C,E). These data show the clear distinction in the limbo-GC identity if caused by the TGF-β or steroid hormone signaling mutations. Ecdysone signaling mutant GSC progeny are kept at the pre-CB stage [(König et al., 2011); Fig. 1B], where chromatin is not yet properly modified to induce the differentiation program (e.g. *bam* expression). *bam* mutant GSC progeny successfully transit through the pre-CB stage (Fig. 1B), modifying chromatin into a ready to differentiate state; however, due to the lack of the major differentiation factor, Bam, these germline cells are not capable of proceeding to the differentiation program. This proposes that *bam* deficient germline cells are blocked in between the pre-CB and CB stage, which we named the “pro-CB” stage (Fig. 1B, GSC→pre-CB→pro-CB→CB). These data suggest that in the hierarchical sequence of events that take place during the transition from stemness to differentiation in the germline, ecdysone signaling operates upstream of Bam. If this is true, then the forced induction of *bam* expression should rescue the delayed differentiation phenotype of ecdysone signaling mutants. We simultaneously perturbed ecdysone signaling and promoted Bam through a combination of heat shock-inducible transgenes, *EcR^hs^* and *bam^hs^*. Forced *EcR* expression negatively regulates ecdysone signaling and causes germline differentiation delay (Schubiger and Truman, 2000; Schubiger et al., 2005; König et al., 2011) and forced *bam* expression leads to stem cell loss by differentiation (Ohlstein and McKearin, 1997) and enhances the germline differentiation index (Fig. 1F). We found that the germline differentiation delay caused by ecdysone signaling deficit was released, if Bam was provided. In *EcR^hs^/bam^hs^* germaria, the differentiation index was significantly increased in comparison to *EcR^hs^* (Fig. 1F). This supports the idea that ecdysone-regulated entrance into the differentiation program operated at the level of chromatin modifications temporally precedes Bam-induced onset of germline differentiation.

Moreover, soma-specific ecdysone signaling perturbation also leads to the H2Bub1 absence in the limbo-GCs showing that ecdysone signaling influences early germline differentiation cell non–autonomously (Fig. 1G). Previously, we and others found that ecdysone signaling is predominantly active in the somatic cells of the germarium (Gancz et al., 2011; König et al., 2011; Morris and Spradling, 2012), here we wanted to decipher the mechanism of how steroids control germline differentiation through the surrounding soma.

### EC function and morphology are impaired by ecdysone signaling perturbations

**Fig. 2.**
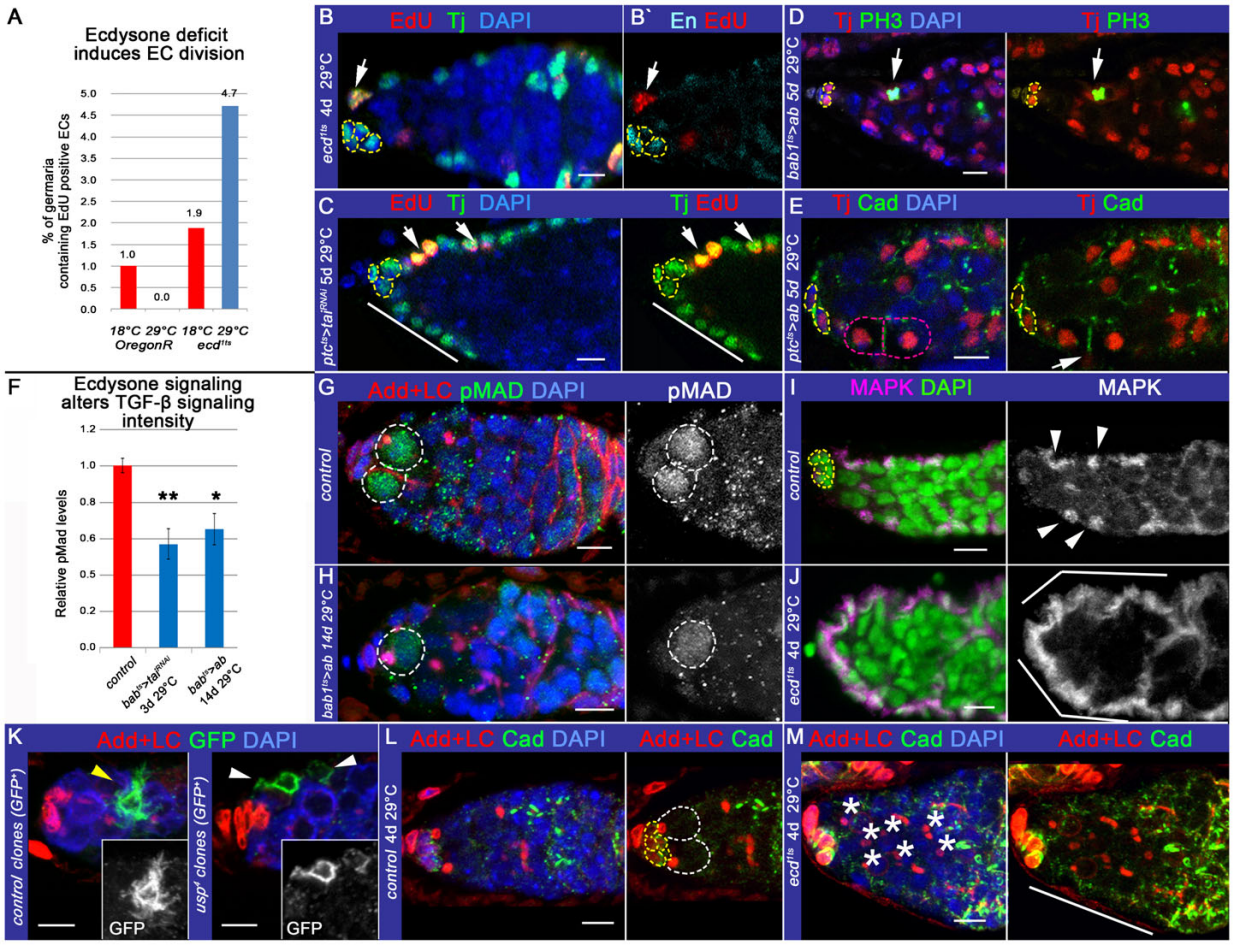
EC function and morphology are impaired by cell-autonomous ecdysone signaling abolishment. (A,B) Perturbed ecdysone signaling induces ECs to divide. In control, less than 2% of germaria exhibit at least one EC in S-phase (*OregonR* 18°C: 1%, n = 99; *ecd^1ts^* 18°C: 1.9%, n = 106), in *ecd^1ts^* adult animals, 4.7% of germaria contain ECs in S-phase (*OregonR*, 4 days at 29°C: 0%, n = 69; *ecd^1ts^* 4 days at 29°C: 4.7%, n = 127). (B–B′) Note the appearance of the EC in S-phase marked by EdU staining. (C–E) Adult specific ecdysone signaling disruption in the soma by *ab* overexpression or *tai* downregulation using the soma-specific *Gal4* drivers (*ptcGal4* or *bab1Gal4*) together with the *Gal4*/*tubGal80^ts^* system alters EC characteristics (*ptc^ts^>tai^RNAi^*: *ptcGal4/tai^RNAi^*; *tubGal80^ts^/+*, *bab1^ts^>ab: tubGal80^ts^/+; bab1Gal4/UASab*, and *ptc^ts^>ab: ptcGal4/+*; *tubGal80^ts^/UASab*, adults kept for 5 days at 29°C). ECs are clustered, resembling columnar epithelia (white line, C). Mutant ECs are also mitotically active; EdU staining shows the presence of ECs in S-phase of the cell cycle (C), and PH3 staining - in M-phase [metaphase (D) and telophase (E)]. (F–H) Upon soma-specific perturbation of ecdysone signaling, the relative pMad levels in the GSCs are decreased. pMad levels are measured by gray value (relative pMad levels, *control: tai^RNAi^/+:* 1±0.04, n = 23; *bab1^ts^>tai^RNAi^: tubGal80ts/+; bab1Gal4/tai^RNAi^*: 0.57±0.08, n = 10, p = 1.48×10^−3^; *bab1^ts^>ab: tubGal80ts/+; bab1Gal4/UASab*: 0.65±0.08, n = 12, p = 7.04×10^−3^). Control (G) and ecdysone signaling defective (H) germaria are shown to compare pMad levels in the GSCs (*OregonR* and *bab1^ts^>ab: tubGal80^ts^/+; bab1Gal4/UASab*, 14 days at 29°C). (I,J) In controls, Map Kinase (MAPK) stains nuclei and clearly defines cytoplasmic protrusions in ECs (arrowheads). In *ecd^1ts^* adults, ECs do not form protrusions and show higher levels of MAPK (*control*: *OregonR*, 4 days at 29°C and *ecd^1ts^*, 4 days at 29°C). (K) MARCM analysis illustrates cytoplasmic protrusions in ECs: clonal ECs are marked by GFP presence. Yellow arrowhead depicts control clonal EC (*w^−^/+*, *FRT 19A tubGal80*, *hsFlp/FRT 19A parental; UAS CD8GFP*, *tubGal4/+*); white arrowheads mark EC homozygous mutant for *usp*, the hetero-dimerization partner of EcR (*w^−^/+*, *FRT 19A tubGal80*, *hsFlp/FRT 19A usp^4^; UAS CD8GFP*, *tubGal4/+*). Note that protrusions of *usp* mutant ECs are less developed in comparison to control. (L,M) High levels of the cell adhesion protein DE-Cad are present on the CpC membranes in the control germarium (*OregonR*, 4 days at 29°C, L). In *ecd^1ts^* (4 days at 29°C, M), higher DE-Cad levels are also detected at the EC membranes. Note that *ecd^1ts^* germaria are filled with a large number of SpGCs delayed in differentiation (asterisks). Germaria are stained with EdU to mark S-phase (red, B,C), PH3 to mark mitotic division (green, D) and LaminC (LC red, G,H,K–M) to visualize TFs and CpCs and Adducin (Add red, G,H,K–M) to mark spectrosomes and fusomes. ECs are at the anterior of the germarium, and are positive for the somatic marker Traffic jam (Tj, green, B, C, red D, E) and negative for Engrailed (En, cyan, B′). EC protrusions are visualized using MAPK staining (magenta, I,J). Cadherin (Cad) marks cell adhesion complexes (green, E,L,M); pMad marks GSCs (green, G,H). ECs were marked with GFP^+^ clones (green, K). Nuclei are marked by DAPI (blue, B–H and K–M, green, I,J). CpCs are outlined in yellow, GSCs in white. Atypical epithelium is highlighted with white lines; mitotically active ECs are marked with white arrows (B–E), EC in telophase is outlined in pink (E). p-values were calculated using the two tailed Student's t-test and error bars represent S.E.M. *p<0.05, **p<0.005, ***p<0.0005. Scale bars, 5 µm.

Ecdysone signaling is important for GSC niche formation during development, but also, for proper EC function during adult stages (Gancz et al., 2011; König et al., 2011; Morris and Spradling, 2012). We examined in a greater detail what happens with the somatic cells in the germaria, depleted of ecdysone signaling during adulthood. Somatic cells in the germarium are responsible not only for germarium architecture organization, mechanical support and physical protection of the germline cells, but they also actively participate in signaling that organizes a microenvironment for proper GSC maintenance and differentiation. For example, the stem cell niche cells (CpCs and, to a lesser extend, ECs) produce TGF-β ligands, required for female germline stemness (Xie and Spradling, 1998; Xie and Spradling, 2000; Eliazer et al., 2014). In contrast, somatic cells in the differentiation niche (ECs) are required to spatially restrict the stem cell niche activity and, as we propose here, to stimulate germline differentiation. Normally, ECs are terminally differentiated squamous epithelial cells that do not divide (Kirilly et al., 2011; Morris and Spradling, 2011); our analysis also revealed that less than 2% of control germaria contained an EC in S-phase marked by EdU (Fig. 2A). In contrast, ecdysone deficit stimulates ECs to proliferate; *ecd^1ts^* flies had 2.5 times more germaria containing ECs in S-phase of the cell cycle (Fig. 2A,B). The specificity of this systemic signaling in the germarial soma is achieved via cell-specific cofactors, Taiman (Tai) and Abrupt (Ab) (König et al., 2011). Tai is a co-activator of the ecdysone receptor complex that can be inhibited by the transcription factor Ab (Bai et al., 2000; Jang et al., 2009). Importantly, EC division phenotype is also seen when ecdysone signaling is perturbed in the soma only: Tai downregulation and Ab upregulation in the somatic cells similarly stimulated normally quiescent ECs to undergo divisions. Mutant ECs at different cell cycle stages were observed (Fig. 2C–E shows cells in S-, meta- and telophase, respectively). Moreover, these changes in EC characteristics were seen when ecdysone production and its signaling co-factors were altered during post-developmental stages (adult-implemented temperature shift for *ecd^1ts^* mutants and induction of temperature sensitive *Gal4/Gal80^ts^* system for adult *tai* and *ab* misexpression). This suggests that the EC destiny as a terminally differentiated squamous epithelial cell is not permanently fixed. Instead, ECs can be transformed under certain conditions during adulthood to exhibit properties of active mitotic division. These cellular alterations could also affect the ability of the cell to properly send and receive signals necessary for early germline maintenance and differentiation.

To prove this assumption, firstly, we tested whether disruption of ecdysone signaling specifically in the stem cell niche cells would affect the TGF-β signaling strength. Both downregulation of ecdysone receptor co-activator *tai* and upregulation of its repressor *ab* in CpCs resulted in a decrease in TGF-β signaling, which could be directly measured via the intensity of antibody staining against pMad. The pMad levels were significantly decreased in GSCs that were located next to the stem cell niche, mutant for ecdysone signaling (Fig. 2F–H). This result demonstrates that upon ecdysone deficit, the GSC niche functions with a reduced efficiency.

Secondly, we tested whether disruption of ecdysone signaling specifically in the differentiation niche cells would affect the strength of the major epithelial pathway, epidermal growth factor receptor (EGFR), that acts in the somatic cells to maintain germline homeostasis. Importantly, compromised EGFR-activated mitogen-activated kinase (MAPK) signaling in ECs results in a similar increase in the SpGC number as is observed in ecdysteroid-deficient germaria (Liu et al., 2010). In ecdysone signaling mutant ECs, MAPK levels were dramatically elevated (Fig. 2I,J; supplementary material Fig. S3A–C), causing aberrant expression of its downstream targets (supplementary material Fig. S3D), which plausibly leads to sexual identity confusion of the somatic cells that compose the GSC and differentiation niches.

The abnormal signaling and behavior causes alterations in EC morphology. While in *controls*, ECs completely enfold germline cells with protrusions to protect differentiating cysts from niche signaling and guide them to the posterior end of the germarium, where the germline becomes encapsulated by the follicular epithelium, in *ecd^1ts^* mutants, these protrusions were not formed and the normally squamous ECs look more like cuboidal epithelial cells outlining the whole germarium (Fig. 2I,J; supplementary material Fig. S3). To visualize EC shape, we also induced MARCM single cell clones and found that in controls, the EC formed multiple delicate protrusions, while the EC, deficient for EcR co-receptor, *ultraspiracle (usp)* lacked these fine cellular structures (Fig. 2K). In addition, ecdysone signaling mutant ECs express higher amounts of cell adhesion molecules, *e.g.* DE-Cad and Arm (Fig. 2L,M; supplementary material Fig. S4), which is consistent with the alteration of their epithelial status. Collectively, these data show that ecdysone signaling deficit is sufficient to alter EC properties: they transform their cell cycle status from quiescent to active and take on a cuboidal rather than a squamous epithelial appearance.

### Levels of Ab, a switch and a manager of epithelial gene expression must be fine-tuned in the germarial soma

**Fig. 3.**
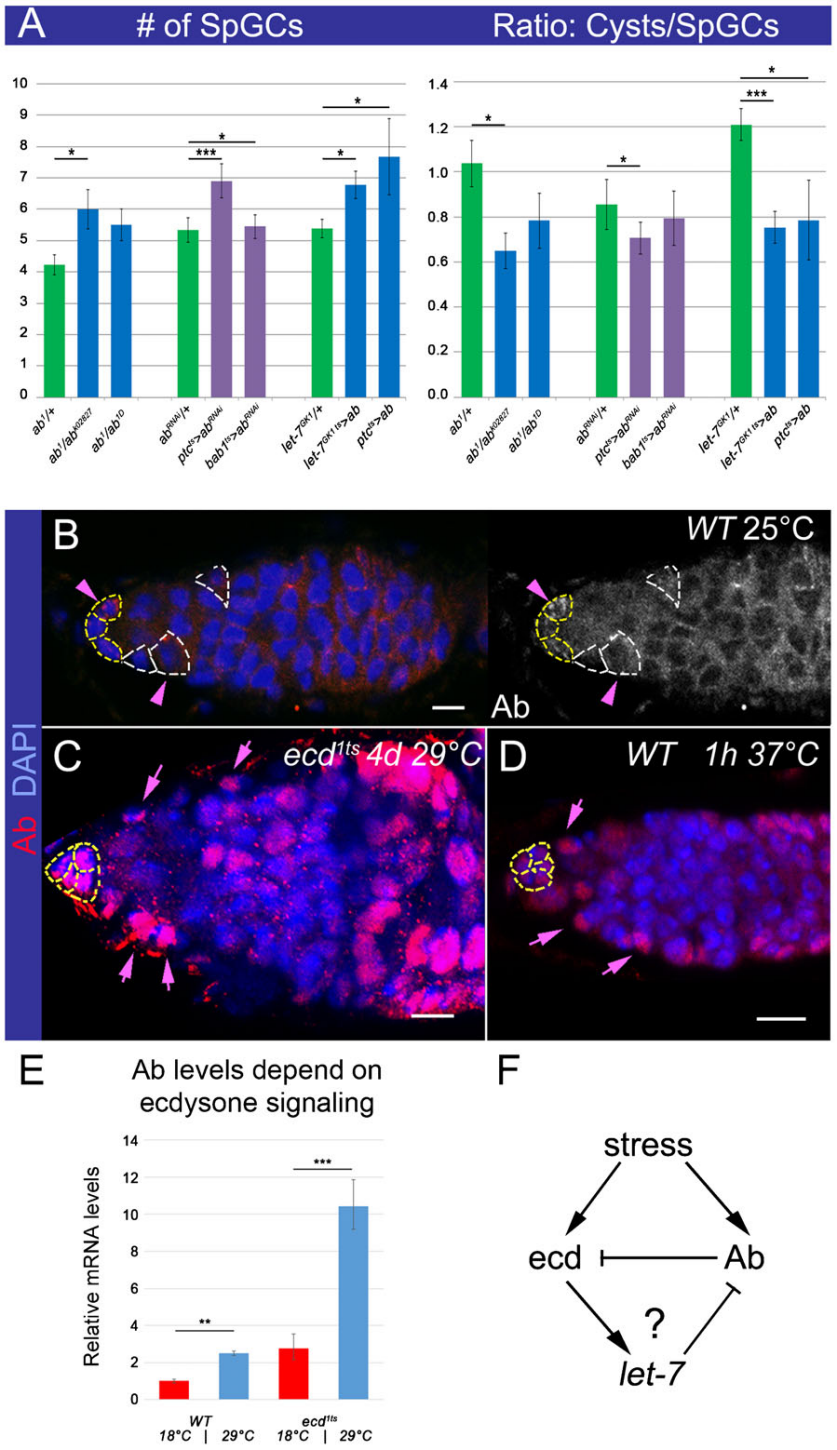
Levels of the transcription factor Abrupt depend on ecdysone signaling and stress and are critical for its subcellular localization. (A) Decreased Ab levels result in the significantly increased number of SpGCs and lower Cysts/SpGCs ratio. Similar effects are observed upon soma-specific *ab* downregulation or overexpression (*ptc^ts^>ab^RNAi^: ptcGal4/ab^RNAi^*; *tubGal80^ts^/+*, *bab1^ts^>ab^RNAi^: tubGal80^ts^/ab^RNAi^*; *bab1Gal4/+*, *let-7^GK1^^ts^>ab: let-7^GK1^/+*; *tubGal80^ts^/UASab* and *ptc^ts^>ab: ptcGal4/+*; *tubGal80^ts^/UASab*, 7 days at 29°C, see supplementary material Table S2). (B) In controls (*OregonR* at 25°C), Ab is only found in the cytoplasm of few somatic cells. ECs are marked with white and CpCs with yellow dashed lines. (C) Ecdysone-depleted or (D) temperature-stressed germaria show strong nuclear Ab staining in the somatic cells. Pink arrows depict strong nuclear and arrowheads cytoplasmic Ab staining in the somatic cells (*ecd^1ts^*, 4 days at 29°C and *OregonR*, 1 h hs at 37°C). (E) The relative Ab mRNA levels are increased in *WT* flies kept at high temperature conditions (*OregonR*, 4 days at 18°C or 29°C). This tendency is even more pronounced in *ecd^1ts^* flies (4 days at 18°C or 29°C, supplementary material Table S3). (F) Hypothetical scheme of the interplay between ecdysone signaling and Ab in response to stress. miRNA *let-7* ensures that the intensity of the steroid hormone ecdysone signaling in adult ovaries is adjusted via downregulation of *let-7* target Ab, which also is a negative regulator of ecdysone signaling. Germaria are stained with Ab (red, B–D), nuclei are marked with DAPI (blue, B–D). p-values were calculated using the two tailed Student's t-test and error bars represent S.E.M. *p<0.05, **p<0.005, ***p<0.0005. Scale bars, 5 µm.

Next, we wanted to understand what molecular mechanism induces transformation of epithelial somatic cells in the germarium and how this transformation can affect early germline differentiation. We turned our attention to the negative regulator of ecdysone signaling, the BTB-zinc finger transcription factor Ab, since it has been found to act even as a potent transdetermination factor that, when misexpressed, is capable of stimulating neuronal identity switch in the developing *Drosophila* brain or induce homeotic arista-leg transformation (Grieder et al., 2007; Kucherenko et al., 2012). In addition, multiple epithelial cell fate regulators have been shown to be direct targets of Ab global transcriptional regulation (Turkel et al., 2013). Similar to other BTB-containing proteins, Ab acts in a highly dosage-dependent manner; depending on the cellular concentration, it can form homo- and heterodimers, which allows for the establishment of both stable and transient interactions with multiple proteins (Hu et al., 1995; Stogios et al., 2005). This explains the ability of BTB-containing proteins to participate in multiple processes and proposes that management of their proper levels is of a particular significance (Kucherenko and Shcherbata, 2013). Previously, we showed that overexpression of Ab in the somatic cells of the germarium recapitulates all of the germline phenotypes associated with a deficit in ecdysone signaling (König et al., 2011). Now, we analyzed the effects of reduced Ab levels using different *ab* amorphic and hypomorphic allelic combinations. Surprisingly, Ab downregulation results in the same phenotypes as its upregulation: the SpGC number was increased, while the ratio between differentiating cysts and SpGCs was decreased (Fig. 3A), confirming that fine–tuning of Ab levels is important for effective early germline differentiation.

An interesting question is therefore, how Ab levels are regulated in the germarial soma and via which molecular mechanisms its expression is coordinated with stress-responsive ecdysone signaling. Previously, it has been shown that Ab represses ecdysone signaling (Jang et al., 2009; König et al., 2011); interestingly, now we found that Ab expression levels itself depend on ecdysone signaling. In ecdysone-deficient flies, *ab* mRNA levels were more than three times higher than in *controls* (Fig. 3E). In addition, Ab protein cellular localization was altered: in *ecd^1ts^* mutant, almost all somatic cells in the germarium exhibited strong nuclear Ab pattern, while in *controls*, only sparse cytoplasmic staining was detected in rare somatic cells (Fig. 3B,C). This pattern changed upon stress; for example, in the wild type germaria analyzed after heat-shock, the majority of the somatic cells contained nuclear Ab, which was present in similar amounts to those found in ecdysone-depleted germaria (Fig. 3B–D). These data show that the BTB transcription factor Ab not only acts as a negative regulator of ecdysone signaling but that its levels are regulated by steroids and stress, which supports the existence of a feedback regulatory loop. Ab was confirmed as a *let-7* target *in vitro* and *in vivo* during pre-adult stages and we found that during metamorphosis, *let-7* miRNA acts as a mediator between Ab and ecdysone signaling in the nervous system (Burgler and Macdonald, 2005; Caygill and Johnston, 2008; Kucherenko et al., 2012). Therefore, we postulated that this regulation might also occur in adult gonads (Fig. 3F).

### Steroid-dependent miRNA *let-7* targets Ab to adjust the ecdysone signaling strength and mediates stress response

**Fig. 4.**
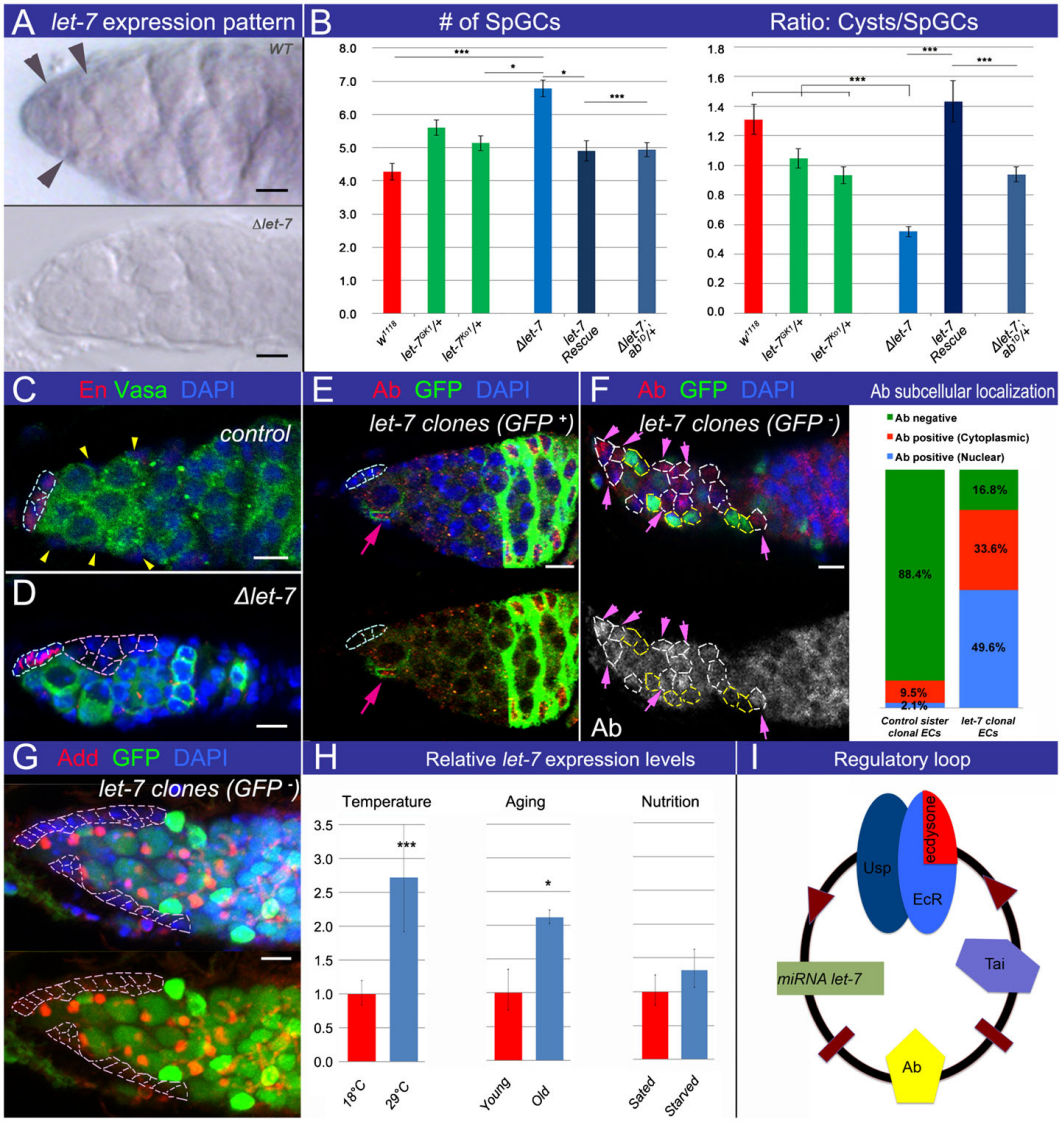
miRNA *let-7* targets Ab to modulate ecdysone signaling response. (A) *let-7* LNA in situ hybridization shows that the mature miRNA is present in ECs of *WT controls* (arrowheads). (B) *let-7* deficiency *(Δlet-7: let-7-C^GK1^/let-7-C^KO1^; let-7-C^Δlet-7^)* leads to delayed early germline differentiation, defined by an increased number of SpGCs and a decreased Cysts/SpGCs ratio. Restoring *let-7* expression (*let-7* Rescue: *let-7-C; let-7-C^GK1^/let-7-C^KO1^*) or reducing Ab levels in the *let-7* deficient background (*Δlet-7; ab^1D^/+: let-7*, *miR-125*, *ab^1D^/let-7^KO1^; let-7-C^Δlet-7^*) rescues this phenotype (supplementary material Table S2). (C,D) In control *OregonR*, single ECs (marked by absence of germline marker Vasa and CpC marker En, depicted with yellow arrowhead) reside along the germarial side. In *Δlet-7* mutants, ECs (marked by the absence of germline marker Vasa and CpC marker En, outlined in pink) clump together. (E) *let-7* MARCM clonal EC (marked by GFP presence, *hsFlp UAS CD8GFP; tubGal80 FRT 40A/let-7 miR-125 FRT 40A; tubGal4/let-7-CΔ^let-7^*) shows high Ab levels (nuclear). (F) The majority of control ECs (GFP-positive, green) are negative for Ab, a high percentage of *let-7* clonal ECs (GFP-negative, black, *hsFlp/+; FRT 40A GFP/FRT 40A let-7 miR-125; let-7-C^Δlet-7^/+*) display cytoplasmic or nuclear Ab. Bar graph shows the percentages of ECs that are Ab-negative or positive for cytoplasmic or nuclear Ab staining. About 50% of *let-7* ECs contain nuclear Ab in comparison to ∼2% in controls (n = 96 and 116 in *let-7* and *control* clonal ECs, respectively). (G) *let-7* clonal ECs (marked by the GFP absence, *hsFlp/+; FRT 40A GFP/FRT 40A let-7 miR-125; let-7-C^Δlet-7^/+*) form the columnar-like epithelium, outlining the anterior tip of the germarium. (H) Germaria, kept at 29°C or aged for 21 days have increased *let-7* levels. Different food conditions do not affect *let-7* levels (see supplementary material Table S4). (I) Scheme showing that in response to stress conditions, *let-7* is capable of generating a sharper threshold response to systemic signaling in the germarial soma via fine-tuning levels of the transcription factor Ab. ECs are located at the anterior of the germarium, and are negative for the CpC marker Engrailed (En, red, C,D) and the germline marker Vasa (green, C,D). Germaria are stained with Abrupt (Ab,E,F). Adducin (Add) marks spectrosomes and fusomes (red, G). GFP presence (E) or GFP absence (F,G) marks *let-7* mutant clones. Nuclei are marked with DAPI (blue, C–G). CpCs are outlined in cyan (C–E). Control ECs are marked with yellow arrowheads (C), *let-7* deficient EC epithelia are outlined in pink (D,G). *let-7* clonal ECs are outlined in white (F) and control sister ECs with yellow (F) dashed lines. Pink arrows indicate *let-7* deficient ECs with nuclear Ab (E,F). p-values were calculated using the two tailed Student's t-test and error bars represent S.E.M. *p<0.05, **p<0.005, ***p<0.0005. Scale bars, 5 µm.

It has been shown before that steroid-coupled regulation of *let-7* expression takes place during the developmental transition from larval-to-reproductive animals and in adult gonads (Sempere et al., 2002; Sempere et al., 2003; Garbuzov and Tatar, 2010; Chawla and Sokol, 2012; Kucherenko et al., 2012; Fagegaltier et al., 2014). To test whether *let-7* is expressed in the correct cell type to act as a transmitter of steroid-modulated response to stress to control oogenesis in adults, we firstly assayed *let-7* miRNA expression. We performed in situ hybridization using *let-7* LNA probe, which confirmed that, like ecdysone signaling components, the miRNA *let-7* is present in the germarial somatic cells (Fig. 4A).

Secondly, we analyzed whether *let-7* loss-of-function would phenocopy the germline phenotypes associated with the ecdysone-signaling deficit. We found that *let-7* deficient mutants, analogously to ecdysone signaling mutants, show retarded germline differentiation that can be restored via exogenous expression of a wild-type *let-7* construct and abnormal EC morphology (Fig. 4B–D). To prove that the *let-7* effect on GSC progeny differentiation is also cell non-autonomous, we induced *let-7* somatic clones and found that clonal ECs, similarly to ecdysone pathway mutants, have a columnar instead of squamous epithelium shape and this affects early germline differentiation (Fig. 4G). Consistent with the somatic *let-7* expression pattern, it does not have a cell autonomous role for GSC maintenance and differentiation (supplementary material Fig. S5A). Since *let-7* loss mimics the phenotypes associated with ecdysone signaling deficit, this implies that ecdysone signaling acts via the miRNA *let-7* to regulate the EC morphology that in turn controls the GSC progeny differentiation.

Thirdly, we analyzed whether Ab is the *bona fide* target of *let-7* in the ECs. We generated adult-induced *let-7* clones and analyzed the expression pattern and cellular localization of Ab protein using Ab specific antibody. *let-7* deficient ECs and follicle cells show high levels of nuclear Ab, indicating that Ab is a *let-7* target in these cells (Fig. 4E). Moreover, Ab overexpression explicitly in *let-7* expressing cells using *let-7* endogenous promoter recapitulates phenotypes associated with ecdysone signaling deficit and results in altered EC shape and delayed GSC progeny differentiation (supplementary material Fig. S4C,D), showing that these two studied components interact in the same cell type. Also, reducing Ab levels by one copy in the *let-7* mutant background significantly rescued *let-7* phenotypes in the germarium (Fig. 4B), confirming the specificity of this regulation in somatic ovarian cells.

Importantly, *let-7* presence in these cells is affecting Ab cellular localization. In control germaria, this transcription factor is largely undetectable; only in ∼10% of ECs a weak cytoplasmic staining was observed. However, of the *let-7* deficient cells, ∼50% exhibit nuclear and ∼30% cytoplasmic Ab staining (Fig. 4E,F). As mentioned before, Ab is a potent regulator of cell fate choices, in order to function as a transcription factor, Ab must localize to the nucleus. Our analyses prove that *let-7* miRNA is capable of targeting Ab in the germarial soma and reveal that in the *let-7* absence, Ab protein levels both increase and undergo altered cellular localization. Recall that nuclear Ab was also seen in ecdysone deficient and stressed wild type ovaries (Fig. 3B–D). Therefore, we propose that upon stress and ecdysone deficit, Ab acts as a regulator of gene expression to adjust function and form of epithelial cells of the germarium, which cell non-autonomously affects the germline differentiation speed. These data are an example of the interesting phenomenon, where transcription factor localization, hence activity, is regulated via miRNAs and steroids in response to stress.

Since Ab subcellular localization is altered due to stress and steroid deficit and ecdysone is considered as a stress hormone (Schwedes and Carney, 2012), we hypothesize that the ecdysone/*let-7*/Ab signaling cascade acts as a modulator of oogenesis in response to different external conditions, where *let-7* is capable of generating a sharper threshold response to systemic signaling in the germarial soma. To test this hypothesis, we analyzed *let-7* levels in the germaria at different stress conditions (heat-shock, aging, and starvation). Interestingly, *let-7* levels were significantly upregulated in the germaria in response to heat stress and aging, but not to malnutrition, suggesting that miRNA *let-7* could be involved in a steroid-dependent adjustment of oogenesis progression in response to some conditions (Fig. 4H). Thus, depending on external conditions, ecdysteroid signaling regulates the early germline differentiation speed via miRNA *let-7* expression induction. Since this miRNA targets a negative ecdysteroid signaling regulator Ab, it suggests a model in which miRNA *let-7* intensifies the ecdysone signaling strength. Notably, stress (for example heat-shock) results in both, an increase in *let-7* expression and activation (nuclear localization) of its target, Ab (Fig. 3B,D and Fig. 4E,F). If there is more of *let-7*, Ab is downregulated and ecdysone signaling is “ON”; if there is more of Ab, ecdysone signaling (and subsequently *let-7*) is “OFF” (Fig. 4I). Thus, *let-7*-modulated adjustment of the ecdysone signaling strength in the germarial soma establishes a certain state of epithelium, defined by the activity of BTB transcription factor Ab. Therefore, we conclude that in the differentiation niche, steroid-induced miRNA *let-7* targets a key epidermal cell fate regulator Abrupt and reinforces ecdysone signaling via a positive feedback loop. We subsequently hoped to probe the mechanism underlying how steroid effects on soma influence germline differentiation.

### Ecdysone signaling modulates differential cell adhesion between the soma and germline that modulates Wg signaling activity in the germline

We next wanted to understand what the nature of this cell non-autonomous signaling between the soma and germline is and when and under what conditions it actually functions. Since (1) Ab is a global regulator of epithelial cell state that is often defined by the specific cell adhesive characteristics and (2) abnormal amounts of cell adhesion proteins, including Cadherin and β-catenin (Fig. 1L,M; supplementary material Fig. S4A,B; Table S3), were detected in ecdysone signaling mutants, we hypothesized that the differential cell adhesion might be a language used for the germline and soma communication. Cadherins normally mediate homophilic adhesion between cells of the same type; however, occasionally cadherins can also be involved in heterotypic adhesion, such as between germline and somatic cells, which occurs across a range of species, including *Drosophila* (González-Reyes and St Johnston, 1998). As a signal transducer, cadherin operates via its binding partner β-catenin, which has a dual role as a mediator in the interplay of adherens junction proteins with the actin cytoskeleton and as a critical Wg signaling pathway component that has been shown to be involved in chromatin remodeling (Liu et al., 2008; Parker et al., 2008; Song et al., 2009; Mohan et al., 2010). A complex non-linear relation between β-catenin and cadherin levels, their subcellular distribution and Wg signaling has been shown in vitro and in vivo (Wodarz et al., 2006; Somorjai and Martinez-Arias, 2008), and proper balance between signaling *vs* adhesive functions is critical for normal development (Gottardi et al., 2001; Brembeck et al., 2006). Since it has been shown that in *Drosophila*, both Wg signaling and cell adhesion mediated by cadherins in the soma play a role in the earliest oogenesis stages (Forbes et al., 1996; Gottardi et al., 2001; Song and Xie, 2003; Brembeck et al., 2006; Sahai-Hernandez and Nystul, 2013) and our data demonstrate that levels of cadherin/β-catenin complexes in the ovarian soma are changed in response to ecdysteroid levels, we tested whether ecdysone signaling via adjustment of cadherin and β-catenin levels can cell non-autonomously modulate the Wg signaling strength in the germline.

**Fig. 5.**
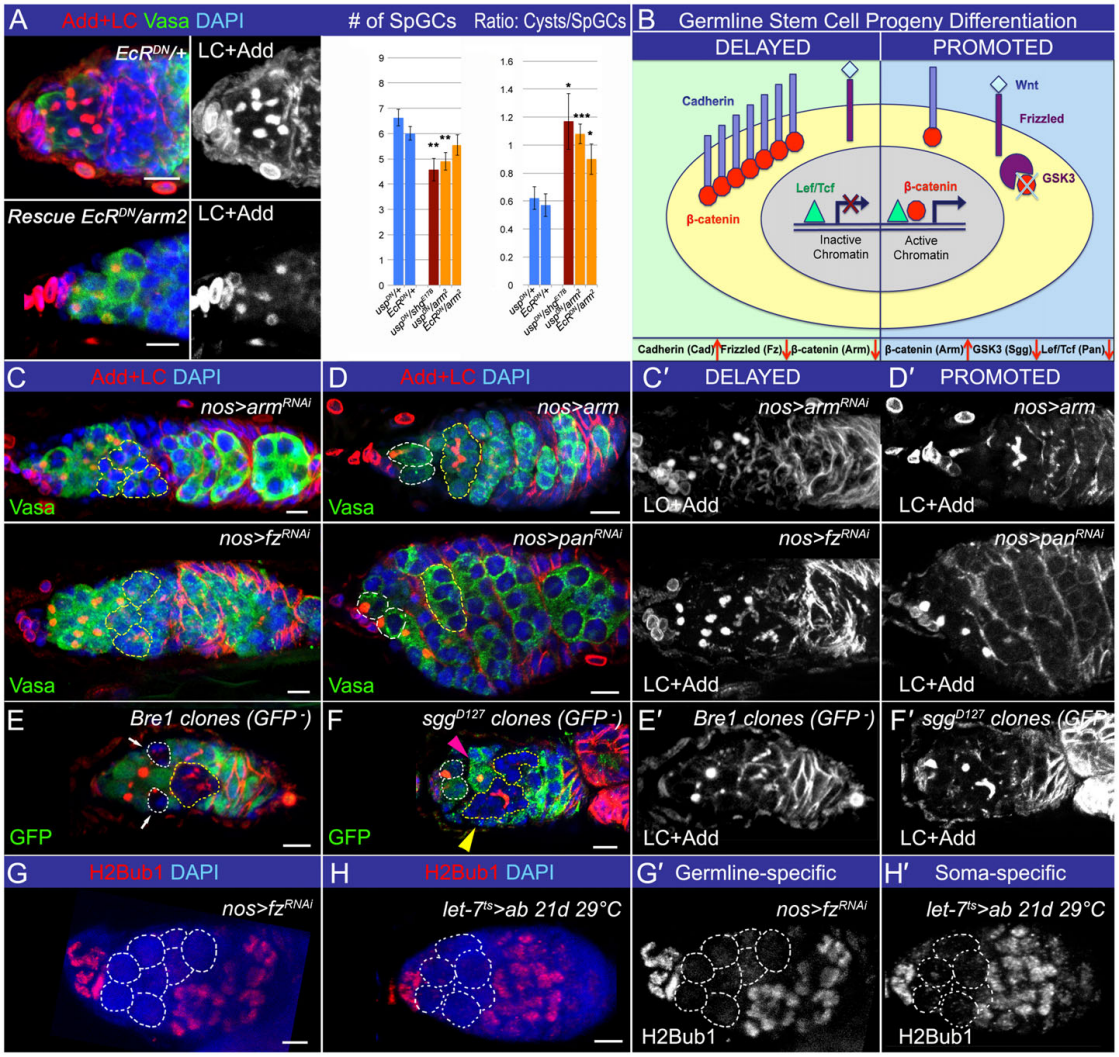
Wg signaling cell autonomously influences the germline differentiation speed. (A) The defects caused by heat-shock induced overexpression of *usp^DN^* or *EcR^DN^* (increased number of SpGCs and decreased Cysts/SpGCs ratio) can be significantly alleviated by reducing the dose of *DE-Cad* or *arm* (*hs-Gal4-usp.LBD/+*, *hs-Gal4-EcR.LBD/+*, *hs-Gal4-usp.LBD/shg^E187^*, *hs-Gal4-usp.LBD/arm^2^* and *hs-Gal4-EcR.LBD/arm^2^*, see supplementary material Table S2). (B) Scheme shows the presumable consequences of Wg signaling perturbation on the germline differentiation speed. (C,C′) Downregulation of Wg signaling activity in the germline (*nos>fz^RNAi^*: *NGT40/fz^RNAi^;nanosGAL4/+* and *nos>arm^RNAi^: NGT40/arm^RNAi^;nanosGAL4/+*) increases the number of SpGCs delayed in differentiation, marked by the presence of the spectrosomes (dot-like Adducin (Add)-positive structures) (see supplementary material Tables S1, S5). (E,E′) Similarly, germline *Bre1* mutant cysts (marked by the absence of GFP, *hsFlp; FRT 2A Bre1^P1549^/FRT 2A GFP*) show delayed differentiation (marked by arrows). Additionally, 10% of germaria with *Bre1* mutant germline cysts contain dying cysts, which was not observed in control (see supplementary material Table S6). (D,D′) In contrast, upregulation of Wg signaling activity in the germline (*nos>pan^RNAi^: NGT40/pan^RNAi^;nanosGAL4/+* and *nos>arm: NGT40/UASarm;nanosGAL4/+*) leads to premature germline differentiation, 8–16-cell cysts are observed already in region 1 of the germarium (see supplementary material Tables S1, S5). (F,F′) The same is observed in *sgg* germline clones (*FRT 101 sgg^D127^/FRT 101 GFP; hsFlp/+*, clones are marked by the absence of GFP). Note that *sgg* clonal germline cells containing a spherical spectrosome (pink arrowhead) and 16-cell cysts (yellow arrowhead) are found side by side (supplementary material Table S6). (G,G′,H,H′) Similar defects in H2Bub1 modification pattern are observed in supernumerary pre-CBs caused by either germline-specific Wg or soma-specific ecdysone signaling perturbations (Fig. 1G). Germaria are stained with LaminC (LC red, A,C–F) to visualize TFs and CpCs and Adducin (Add red, A, C–F) to mark spectrosomes and fusomes. Vasa marks germline (green, A,C,D). Absence of GFP (green, E,F) marks clonal mutant cells. Monoubiquitination of H2B is shown (red, G,H). Nuclei are marked with DAPI (blue, A,C–H). White dashed lines mark GSCs (D,F), differentiation delayed GCs (E) or GSCs and additional SpGCs (G,H). Yellow dashed lines depict differentiating cysts (C,D) or clonal mutant differentiating cysts (E,F). p-values were calculated using the two tailed Student's t-test and error bars represent S.E.M. *p<0.05, **p<0.005, ***p<0.0005. Scale bars, 5 µm.

Our data show that increased DE-Cad levels in ecdysone signaling-deficient ECs correlate with pre-CB differentiation delay (Fig. 2L–M; supplementary material Fig. S4). Importantly, we found that soma-specific reduction of DE-Cad or Arm levels in ecdysone mutant background rescues early germline differentiation phenotypes (Fig. 5A), while overexpression of DE-Cad in ECs phenocopies ecdysone signaling phenotype (supplementary material Fig. S4E); together demonstrating that ecdysone signaling and DE-Cad/Arm interact in the ECs. This observation suggests that readjustment of cadherin and β-catenin levels can mediate ecdysone-dependent cell adhesive characteristics that influence the germline differentiation progression.

One implication for this cell non-autonomous signaling is that in response to increase in DE-Cad in the soma, more DE-Cad would be recruited to adherens junctions in the germline. This would affect the cellular Arm distribution in the germline cells, tethering it to the cell membrane to be associated with DE-Cad (Fig. 5B). Normally, in the absence of Wg ligands, the cytoplasmic β-catenin pool is easily degraded by a complex containing APC, CKI, Axin, and GSK3/Shaggy(Sgg) (Kim et al., 2013). Upon Wg ligand binding to its receptors, such as Frizzled (Fz), a protein called Dishevelled (Dsh) is activated and leads to the Arm degrading complex inactivation. When the Arm degradation is inhibited, it allows its entrance to the nucleus, where Arm forms a complex with the Lef/TCF/Pangolin(Pan), activating the transcription of target genes (Chien et al., 2009). Without Arm, Lef/TCF/Pan together with their cofactors recruit histone deacetylases, triggering chromatin modifications that promote transcriptional silencing (Saito-Diaz et al., 2013). Upon Arm binding, multiple co-activators involved in chromatin remodeling are recruited, which stimulates transcription (Valenta et al., 2012). Since Arm pools involved in cell adhesion and Wg signaling exchange, we propose that differential cell adhesion between the germline and somatic cells affects the Wg signaling activity in the germline that regulates the GSC progeny chromatin state. If this hypothesis is right, we would expect that genetic manipulations that downregulate Wg signaling in the germline would delay, while Wg signaling overactivation would promote early germline differentiation (Fig. 5B).

To test this, we first examined whether Wg signaling is acting in the germline by specific Wg pathway components targeting in the germline, using recently created VALIUM20 *UAS RNAi* library and the germline specific *nanosGal4* driver. Downregulating Wg signaling *(UASfz^RNAi^*, *UASarm^RNAi^)* resulted in the appearance of huge ecdysoneless-like germaria, containing supernumerary limbo-GCs indicative of delayed differentiation (compare Fig. 5C and Fig. 1D, Fig. 2M, Fig. 4C). In contrast, increasing Wg pathway activity *(UASarm*, *UASsgg^RNAi^*, *UASpan^RNAi^)* accelerated germline differentiation. Precociously differentiating 8–16 cell cysts were seen already in region 1 (instead of 2B) of the germarium (Fig. 5D). Similar phenotypes were observed when germline clones lacking *arm* and *sgg* were analyzed; 6–13% of clonal germaria contained *arm* germline cysts delayed in differentiation, while 47% of clonal germaria contained *sgg* germline cysts precociously differentiating, as judged by the differentiation stage of non-clonal neighbors (Fig. 5F; supplementary material Table S6). These data show that Wg signaling acts in the germline to cell autonomously influence the early germline differentiation tempo. Similarly to Wg downregulation, germline-specific perturbations of H2Bub1 modification *(UASBre1^RNAi^* and *UASRtf1^RNAi^)* influenced the efficiency of germline differentiation (supplementary material Fig. S2; Table S1). Also, 20% of *dBre1* clonal germaria contained cysts that were delayed in differentiation (Fig. 5E; supplementary material Table S6). Since Wg signaling has been shown to interact with chromatin remodelers (Liu et al., 2008; Parker et al., 2008; Song et al., 2009), we analyzed whether perturbed Wg signaling would affect the chromatin status of the germline cells. As expected, and in contrast to the cell non-autonomous function of steroid signaling, Wg signaling controls this process cell autonomously, since its germline-specific perturbation results in the H2Bub1-negative pre-CB accumulation (Fig. 5G,H). This supports the idea that intrinsic Wg signaling controls germline differentiation at the level of chromatin modification.

**Fig. 6.**
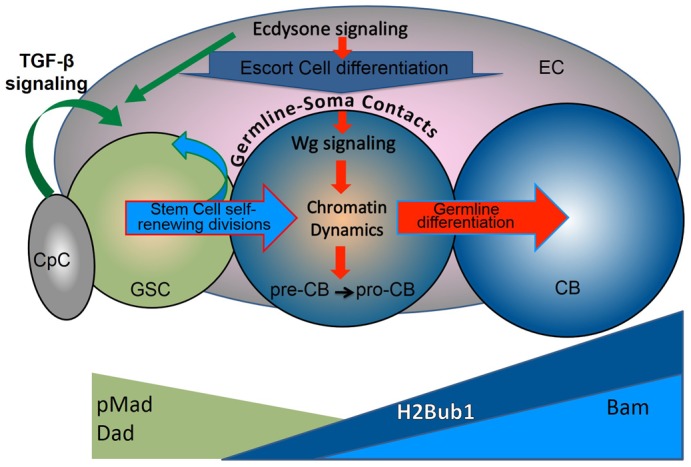
Model showing ecdysone signaling function in early germline differentiation. The efficiency of early germline differentiation is managed by multiple proteins involved in different signaling pathways, which act in both the germline and soma. The GSC progeny differentiates in a sequential manner via specific transitional steps (GSC→pre-CB→pro-CB→CB) that are easily identified by the expression patterns of the specific markers. Importantly, these transitions between the germline differentiation stages in the germarium are controlled by signaling from the surrounding soma. As the stem cell niche (CpCs) controls the stemness of GSCs, the differentiation niche (ECs) coordinates the efficiency of GSC progeny differentiation with the status of the whole organism via systemic signaling. The GSC maintains its stem cell characteristics due to TGF-β signaling from the stem cell niche. Upon GSC division, its daughter is pushed away from the stem cell niche and, therefore, cannot receive sufficient TGF-β signaling to support the stem cell identity. However, being away from the niche is not enough to start the differentiation program. As the GSC progeny (pre-CB) is detached from the niche, its adhesive connection to the soma weakens; Cad levels are reduced. This releases Arm (β-catenin) from binding to Cad; instead, more Arm becomes available for Wg signaling, as Arm is not only involved in the cell adhesion in complex with Cad, but also, it is the transducer of Wg signaling. As the germline and soma are connected via homophilic cell adhesion, reduction of Cad levels results in strengthening of Wg signaling in the germline. Combination of decreased TGF-β and increased Wg signaling in the germline promotes chromatin modifications (e.g. H2Bub1) from the stem cell state into the differentiation-ready state, which endorses expression of the differentiation genes. The cell becomes the pro-CB, the key differentiation factor Bam begins to be expressed, initiating the differentiation processes in the germline. Importantly, the efficiency of these early germline differentiation events is modulated by systemic signaling. Particularly, ecdysone signaling cell autonomously alters somatic cell characteristics in the differentiation niche in response to stress, affecting adhesive contacts between the soma and germline, which, in turn, influences Wg signaling in the germline (CB). Thus, steroids cell non-autonomously affect the early steps of germline progression to coordinate the germline differentiation speed with the status of the whole organism.

Together these data demonstrate a communication flow, where the signal from the soma (ecdysone signaling-cell adhesion) induces in the germline a transition from stemness to differentiation, which requires a specific histone modification that promotes the expression of differentiation genes, enabling efficient GSC progeny differentiation (cell adhesion-Wg signaling-H2Bub1-Bam) (Fig. 6). Importantly, ecdysone and Wg signaling alterations or H2Bub1 histone modification do not cause a complete germline differentiation block, just a delay. This supports the idea that described here mechanism provides an additional layer of adult oogenesis regulation that is used to fine-tune the GSC progeny differentiation tempo in response to organism fitness fluctuations caused by internal and external environment changes.

## DISCUSSION

In summary, our results show a cooperative function between hormonal steroid and Wg signaling in the fine-tuning of the early germline differentiation speed in response to variations in the organism physiology and environmental conditions. Ecdysone signaling robustness is conferred via the *let-7* miRNA feedback loop: *let-7* expression is ecdysone–dependent, while the *let-7* target Ab is a negative ecdysone signaling regulator. This allows upon *let-7* expression to enhance the steroid signaling strength, which then cell autonomously adjusts EC characteristics and cell non-autonomously influences the early germline differentiation speed. This regulation occurs via differential cell adhesion between the germline and soma that, in turn, modifies the Wg signaling strength in the germline (Fig. 6). The balance in Wg pathway activity is important for normal germline differentiation, since Wg overactivation stimulates premature germline differentiation, while Wg downregulation slows-down the process. These data for the first time reveal that Wg signaling has cell autonomous role in the *Drosophila* germline. It is important to stress that neither Wg signaling in the germline, nor ecdysone signaling in the soma are absolutely required for germline progeny differentiation, both pathways just add an additional layer of regulation, possibly interacting with other pathways, which would result in formation of a complex regulatory network that fine-tunes the efficiency of egg maturation in accordance with alterations in the external and internal environment of the organism.

Multiple studies propose that the GSC progeny differentiates by performing an orderly sequence of steps that are defined by expression of multiple factors (Fig. 6). The GSC maintains its stem cell identity due to signaling from the niche. Its daughter is pushed away from the niche, does not receive the sufficient amount of stem cell niche signaling and is transformed into the pre-CB. The pre-CB starts to modify its chromatin and becomes committed to differentiate, turning into the pro-CB. The pro-CB has all the conditions necessary for expression of differentiation genes. The key differentiation factor starts to be expressed and the cell is transformed into the CB. Thus, complex sequences of cellular transformation steps are required to produce the mature egg, many of which are already happening in the germarium and influenced by the surrounding soma that assembles the stem cell and the differentiation niches that can sense and transduce the extracellular signals to coordinate the tempo of germline differentiation.

Principally, our data demonstrate that oogenesis is a highly regulated process that depends on external and internal status of the female and that systemic signaling coordinates the oogenesis speed depending on these conditions. Steroids act in the soma and cell non-autonomously and, via the direct cell adhesion-based soma-germline communication, govern germline differentiation. If conditions are unfavorable, insufficient steroid signaling can delay the GSC progeny in the pre-CB state, meaning that the cell is caught in the in-between stem cell and differentiating cell transition. This delayed cell does not receive the stem cell niche signaling anymore (because it is detached from the niche), but it also does not receive the signal to differentiate that, as we propose here, is generated in the differentiation niche (ECs) in response to ecdysone signaling. This signal is extremely dosage-dependent and, as we show here, multiple pathways can add to its implementation (supplementary material Fig. S6). Biologically, this makes sense, since this information controls the reproductive efficiency and thus, the success of organism survival. However, steroids are not the sole regulators of the process. It is known that oogenesis is an energy demanding process and strongly depends on the nutritional state. Kept on rich food, flies produce 60 times more eggs than those on poor food (Drummond-Barbosa and Spradling, 2001). Insulin signaling and other pathways were shown to mediate the response to food availability; GSCs and FSCs respond to the nutritional conditions by adjusting their proliferative rate (Biteau et al., 2010). Two systemic signaling pathways, insulin and ecdysone regulate the oogenesis efficiency, however act upon different cell types and germline progression stages. Interestingly, upon starvation, oogenesis is blocked at stage 8 and most of the dramatically decreased egg production rate seems to result from an increased degeneration of later egg chambers (Drummond-Barbosa and Spradling, 2001). Ecdysone is produced in older follicles that passed the stages 8–9 and is required to progress past this master checkpoint (Buszczak et al., 1999; Carney and Bender, 2000). Thus, there are two independent checkpoints that control the oogenesis progression: at stage 8, an insulin-dependent or “nutritional checkpoint” that influences the GSC division speed, and at stage 8–9 an ecdysone-dependent or “stress checkpoint” that influences the GSC progeny differentiation speed. Ecdysone control of germline development, therefore, presents a positive feedback mechanism with which germline development in the germarium is synchronized with the presence or absence of older follicles. Together, orchestrated regulation of oogenesis executed by insulin and ecdysone hormones reassures that the germline differentiation speed is perfectly attuned to external and internal cues.

The cell is what it is because it expresses a certain combination of genes that establish its form and function. Not surprisingly, control of the quantity and quality of gene expression is key for the cell morphology establishment and cellular signal transduction. We show that in the ovary, the differentiation niche, comprised of specifically shaped ECs exists and that the shape of these cells regulates the differentiation ability of the neighboring GSC progeny. The EC shape and function are dramatically impaired in ecdysone signaling mutants: the squamous ECs line the germarium and form long cytoplasmic protrusions that envelop the developing germline cells. However, if ecdysone signaling is perturbed, ECs form layers that resemble columnar epithelium. In addition, thin cytoplasmic protrusions are no longer present and the levels of cell adhesion proteins are elevated. Earlier experiments already demonstrated how critical EC protrusions are: if the protrusion formation is inhibited specifically in the ECs – for example via interfering with the cytoskeleton – the germline differentiation is affected and a larger number of SpGCs was detected (Decotto and Spradling, 2005; Wang et al., 2008; Kirilly et al., 2011). A lack of EC protrusions therefore can cause a differentiation delay in the germline and we propose that EC malformation, and specifically the absence of protrusions, contributes to the differentiation delay observed in the germline. How exactly EC protrusions enable germline differentiation has previously been poorly understood; it was thought that they physically shield differentiating germline cells against diffusible signals from the anterior CpCs, forming another barrier to locally restrict TGF-β signaling. Now we show that the EC's ability to form protrusions depends on levels of cell adhesion proteins. Due to homophilic cell adhesion rules, alterations in cell adhesion protein quantities and qualities in the soma lead to the readjustment of these in the adjacent germline, as cadherin levels must perfectly match, this information is conveyed to the germline. Cell adhesion proteins are not only involved in establishment of cell connections, they also participate in intercellular signaling (Edeleva and Shcherbata, 2013), particularly, β-catenin is the binding partner of a homophilic cell adhesion receptor DE-Cad and the effector of the canonical Wg/Wnt signaling pathway (MacDonald et al., 2009). Since β-catenin pools involved in both processes are interchangeable, an increase or decrease in β-catenin involvement in cell adhesion would affect pools available for Wg signaling, subsequently readjusting the Wg pathway efficiency (Valenta et al., 2012). This pathway is one of the key developmental pathways that, via its interaction with the histone modification machinery, globally regulates gene expression (Saito-Diaz et al., 2013). Therefore, by simply changing one cell type's morphology, the transcriptional status of the juxtaposed cell could be affected. This kind of regulation is especially attractive for communication between cells of different types that are joining together to build a tissue or an organ and have to coordinate their signaling in order to function in unison. In particular, such heterotypic cell interaction modes are interesting in the regulation of stem cell maintenance and stem cell progeny differentiation via niches in response to systemic organismal demands.

## Supplementary Material

Supplementary Material
