# Supplementary material for: Soma influences GSC progeny differentiation via the cell adhesion-mediated steroid-let-7-Wingless signaling cascade that regulates chromatin dynamics
Source: Biol Open. 2015 Feb 6;4(3):285–300. doi: 10.1242/bio.201410553 (PMC4359735; doi:10.1242/bio.201410553)
Supplement: Supplementary Material [file supp_4_3_285__index.html]

Soma influences GSC progeny differentiation via the cell adhesion-mediated steroid-let-7-Wingless signaling cascade that regulates chromatin dynamics — Supplementary Material 

# Soma influences GSC progeny differentiation via the cell adhesion-mediated steroid-*let-7*-Wingless signaling cascade that regulates chromatin dynamics

## bio.201410553 Supplementary Material

**Files in this Data Supplement:**

- Supplementary Material - Annekatrin König and Halyna R. Shcherbata doi: 10.1242/bio.201410553
