# Supplementary material for: Soma influences GSC progeny differentiation via the cell adhesion-mediated steroid-let-7-Wingless signaling cascade that regulates chromatin dynamics
Source: Biol Open. 2015 Feb 6;4(3):285–300. doi: 10.1242/bio.201410553 (PMC4359735; doi:10.1242/bio.201410553)
Supplement: Supplementary Material [file supp_bio.201410553_bio.201410553-s1.pdf]

Supplementary Material
Annekatrin König and Halyna R. Shcherbata doi: 10.1242/ bio.201410553

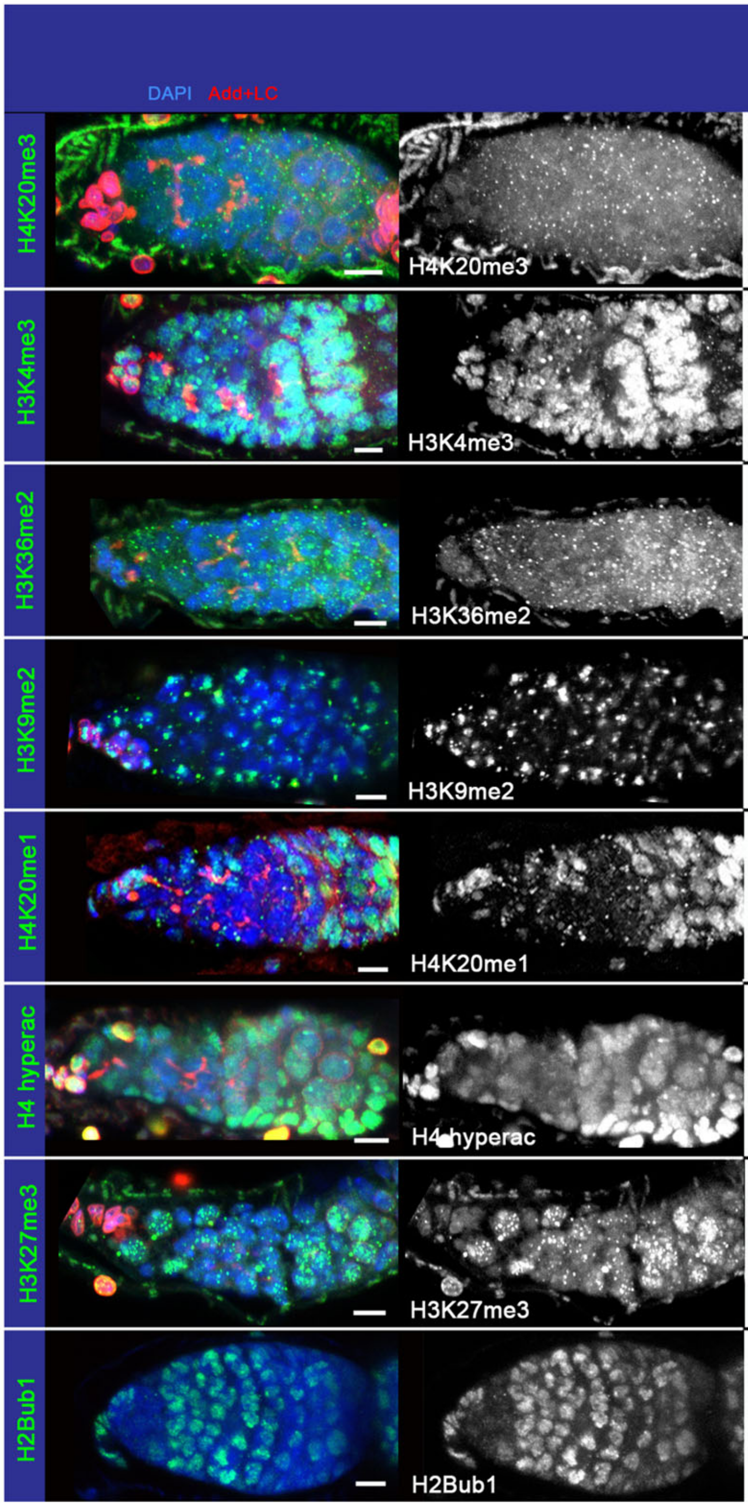

	Soma			Germline			
	CpC	EC	FC	GSC	CB	4/8 cell cyst	16/cell cyst
H4K20me3	+	+	+	+	+	+	+
H3K4me3	+	+	+	+	+	+	++
H3K36me2	+	+	+	+	+	+	++
H3K9me2	++	++	+	+	+	+	+
H4K20me1	++	+	++	+	+	+	++
H4 hyperac	++	+	++	+	+	+	+
H3K27me3	+	+	+	++	++	++	+++
H2Bub1	++	+	+	-	++	++	++

Fig. S1. Histones are differentially modified in somatic and germline cells in the germarium. Wildtype (*OregonR*) germaria are stained with several histone modification antibodies (green); in addition, germaria are stained with LaminC (LC red) to visualize TFs and CpCs and Adducin (Add red) to mark spectrosomes and fusomes. Nuclei are marked with DAPI (blue). In particular, H4K20me3, H3K9me3, H3K9me2, H3K27me3 and H4K20me1 are associated with transcriptional repression, while H3K4me3, H4 hyperacetylation, H2Bub1 are known as active marks and often associated with ongoing transcription. Note that some histone modifications show differential pattern in certain somatic cells and differentially staged GCs; importantly, H2Bub1 is present in the differentiating cysts, but not in the GSCs. Scale bars, 5 µm.

Biology Open (2015) 000, 1–16 doi:10.1242/bio.201410553

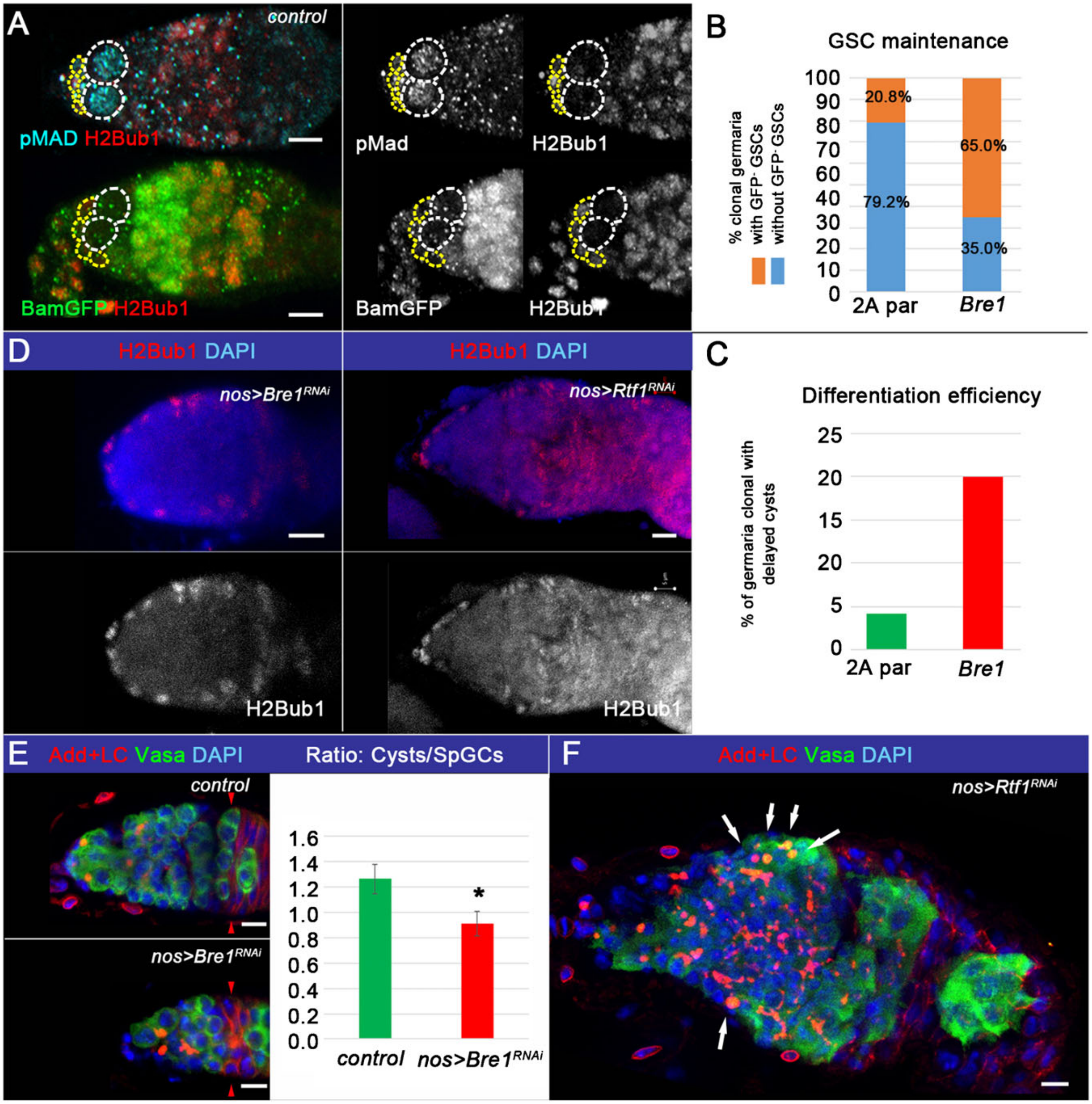

Fig. S2. *H2Bub1*-deficient germline cysts are delayed in differentiation. (A) In the *control (OregonR)* germarium, GSCs exhibit pMad staining. The differentiation marker Bam (detected by the *bamGFP* transgene) is expressed in cysts. H2Bub1 is found in cysts that are also positive for Bam, but not in GSCs. (B,C) *Bre1* mutant (*hsFlp; FRT 2A Bre1*^{P1549}*/FRT 2A GFP*) and control (*hsFlp; FRT 2A parental/FRT 2A GFP*) germline clones are analyzed 5 days after adult clone induction. Germaria containing *Bre1* germline clones frequently lack *Bre1* clonal GSCs; bar graph shows that 79.2% (n=24) of control germaria contain one or more black GSCs, while *Bre1* clonal GSCs are partially lost and only 35.0% (n=20) of all clonal germaria contained one or more black GSCs (B; see supplementary material Table S6). (C) *Bre1* clonal cysts are delayed in differentiation. 4.16% (n=24) of control and 20% (n=20) of *Bre1* germline clonal cysts show the differentiation delay. (D) Analysis of H2Bub1 modification upon downregulation of Rtf1 or Bre1 using RNAi shows that Bre1 and Rtf1 are specifically required for monoubiquitination of H2B in the germline, since their downregulation (*nos*>*Bre1*^{RNAi}*: NGT40/Bre1*^{RNAi}*;nanosGAL4/+* and *nos*>*Rtf1*^{RNAi}*: NGT40/Rtf1*^{RNAi}*; nanosGAL4/+*) results in the absence of this modification. (E,F) Germline-specific downregulation of Bre1 affects differentiation (*control: NGT40/+;nanosGAL4/+* and *nos*>*Bre1*^{RNAi}*: NGT40/Bre1*^{RNAi}*; nanosGAL4/+*); (E) reducing Bre1 levels in the germline leads to the appearance of small germaria (red arrowheads mark beginning of region 2b) and the decrease in the differentiation index (Cysts/SpGCs, supplementary material Table S1). (F) Expression of *Rtf1*^{RNAi} in the germline (*NGT40/+;nanosGAL4/Rtf1*^{RNAi}) leads to severe perturbations of germarial architecture and defects in germline differentiation; SpGCs are found at arbitrary positions (arrows) far from the stem cell niche. pMad marks GSCs (cyan, A), bamGFP (green, A) differentiating cysts. Monoubiquitination of H2B is shown (red, A,D), germline cells are Vasa-positive (green, E,F). Germaria are stained with LaminC (LC red, E,F) to visualize TFs and CpCs and Adducin (Add red, E,F) to mark spectrosomes and fusomes. Nuclei are marked with DAPI (blue, D–F). GSCs are outlined in white, CpCs in yellow (A). H2Bub1-positive CpCs are marked with white arrows (D), H2Bub1-positive ECs with yellow arrows (D). Region 2b is indicated by red arrowheads (E) and SpGCs are depicted with white arrows (F). p-values were calculated using the two tailed Student's t-test and error bars represent S.E.M., *$p<0.05$. Scale bars, 5 µm.

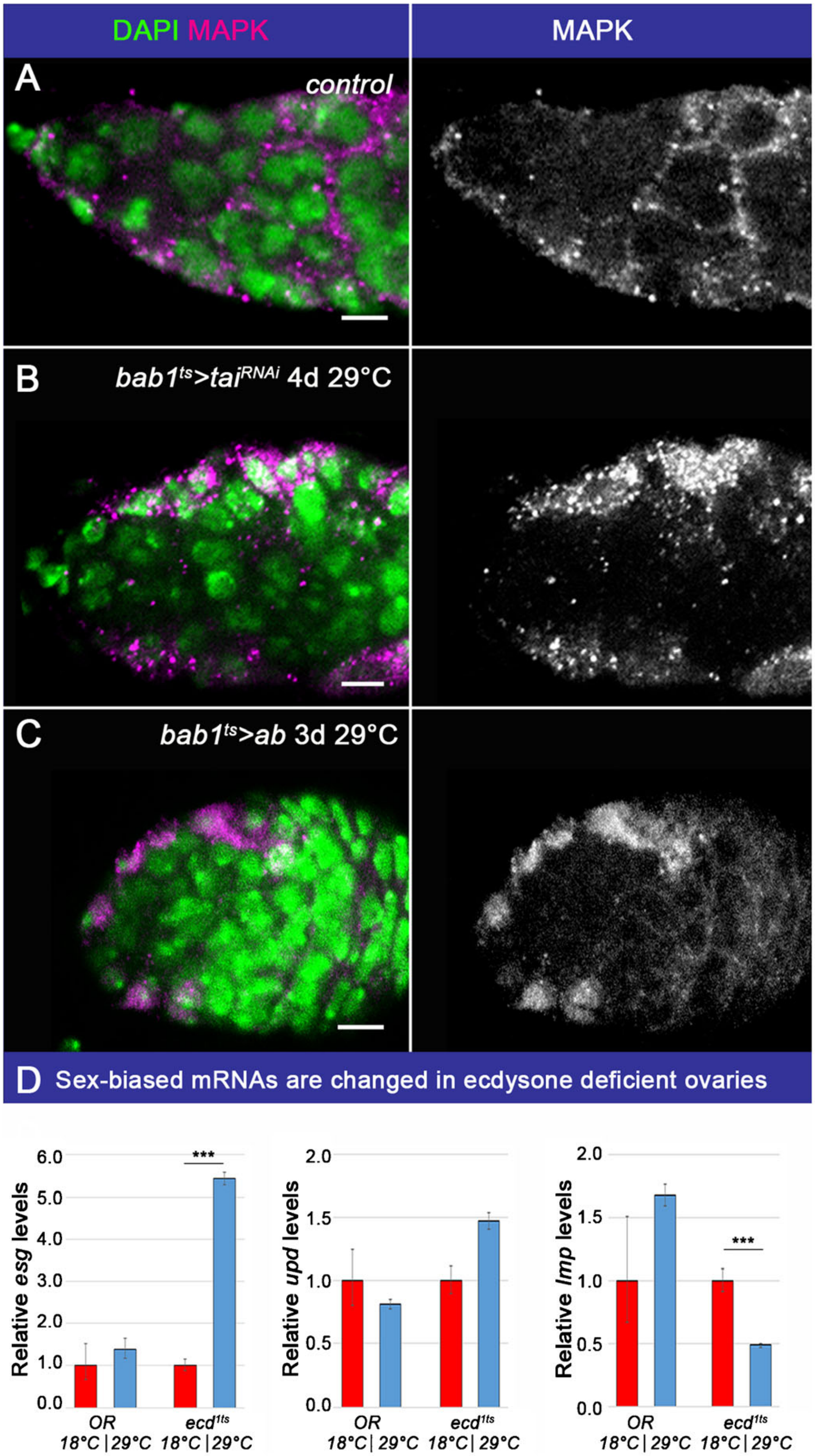

Fig. S3. EC cellular identity is affected upon soma-specific perturbation of ecdysone signaling. (A) ECs form long cytoplasmic protrusions with which they envelope the differentiating germline (*control*: *tubGal80*^{ts}/+ ; *bab1Gal4*/+). (B,C) Somatic alteration of ecdysone signaling during adulthood dampens the ability of ECs to form protrusions. The *Gal4/Gal80*^{ts} system was used to express *tai*^{RNAi} or *ab* using *bab1Gal4* somatic driver in adults (*bab1*^{ts}>*tai*^{RNAi}: *tubGal80*^{ts}/*tai*^{RNAi}; *bab1Gal4*/+, 4 days at 29˚C and *bab1*^{ts}>*ab*: *tubGal80*^{ts}/+; *bab1Gal4/UASab*, 3 days at 29˚C). MAP Kinase (MAPK) staining shows cytoplasmic protrusions in ECs (magenta). Note that MAPK levels are increased in ecdysone signaling-deficient ECs (D). Ecdysone signaling affects the maintenance of cellular sexual identity in adult *D. melanogaster* gonads (Fagegaltier et al., 2014) and multiple EGFR-MAPK downstream targets are sexually biased. mRNA levels of direct and indirect EGFR-MAPK signaling pathway downstream targets, *escargot (esg)*, *unpaired (upd)* and *IGF-II mRNA-binding protein (Imp)* (López-Onieva et al., 2008; Chau et al., 2009; Toledano et al., 2012) are deregulated. In *ecd*^{1ts} germaria, the expression levels of the male-specific mRNA *esg* increase ~5 fold and the mRNA levels of JAK/STAT ligand *upd* ~1.5 fold, while the levels of *Imp* decrease ~2 fold (supplementary material Table S3). These data show that upon defective ecdysone signaling, EGFR signaling is impaired, resulting in confused sexual identity in the somatic cells of the germarium. MAPK staining marks ECs and EC protrusions (magenta, A–C), DAPI marks nuclei (green, A–C). ***$p<0.0005$. Scale bars, 5 µm.

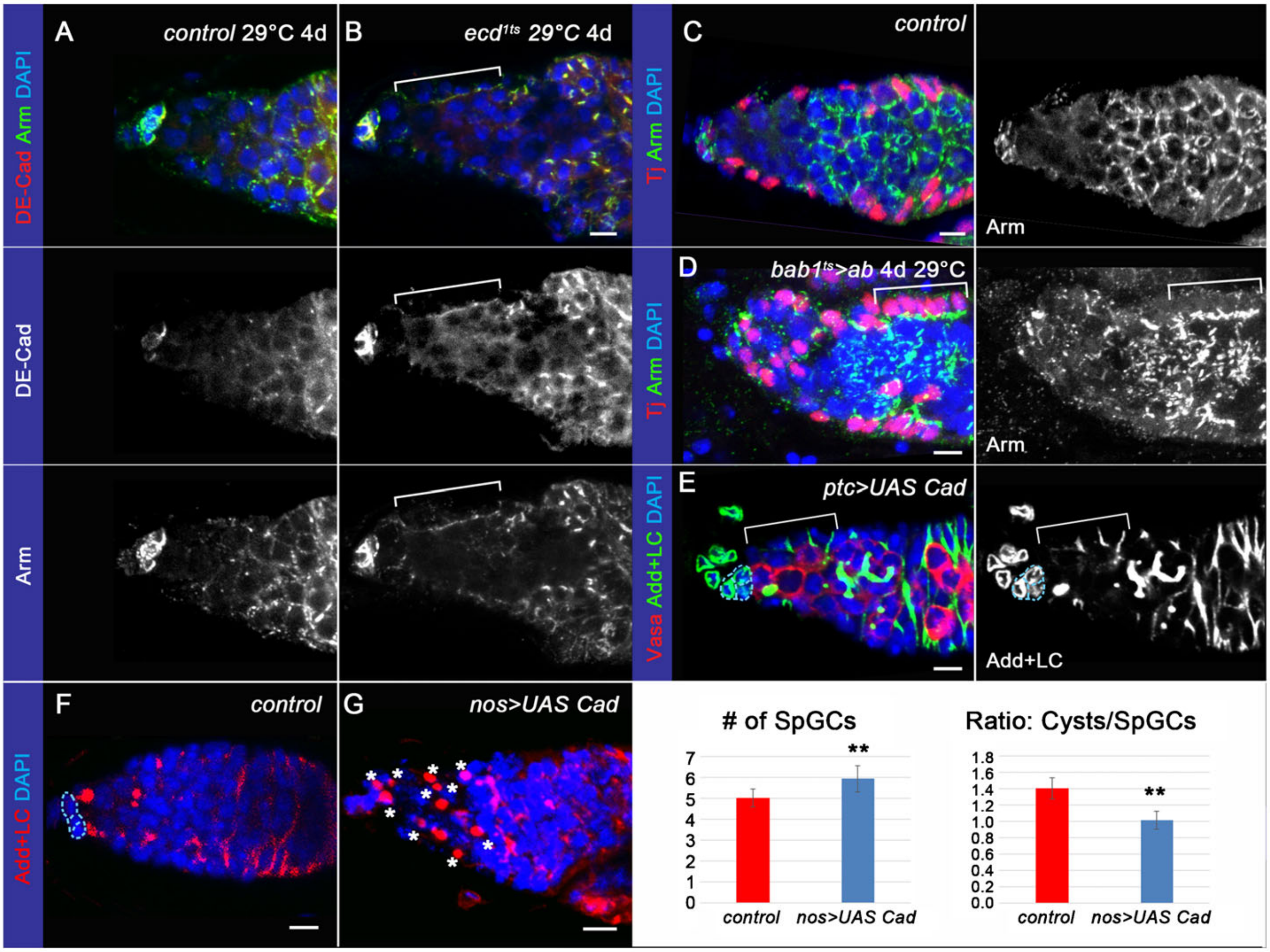

Fig. S4. Soma-specific disruption of ecdysone signaling causes altered cell adhesion between ECs and the germline. (A,B) High levels of the cell adhesion proteins DE-Cad and Arm are detected at the membrane of CpCs (*control: OregonR*). In *ecd^{1ts}* adult flies kept at the restrictive temperature (29˚C) for four days, high levels of Arm and DE-Cad are also found on the EC membrane (marked with brackets). (C,D) Downregulation of ecdysone signaling in the soma by overexpressing its negative regulator Ab with the soma-specific driver (*control*: *tubGal80^{ts}/+*; *bab1Gal4/+* and *bab1^{ts}>ab: tubGal80^{ts}/+*; *bab1Gal4/UASab*, 4 days at 29˚C) results in the increased Arm levels in the ECs, atypical ECs form epithelial layers (bracket). (E) Similarly, overexpression of DE-Cad with the somatic driver (*ptc/UAS Cad*) leads to formation of EC epithelial layers (bracket). (F,G) Germline-specific overexpression of Cad (*control: NGT40/+; nanosGAL4/+* and *nos>Cad: NGT40/UAS Cad;nanosGAL4/+*) leads to a higher number of SpGCs (asterisks) and an increased Cysts/SpGCs ratio (supplementary material Table S1). Cell adhesion complexes are marked by DE-Cad (red, A,B) and Arm (green, A–D). ECs are positive for Tj (red, C,D) and negative for the germline marker Vasa (red, E). Germaria are stained with LaminC (LC red, F,G, green, E) to visualize TFs and CpCs and Adducin (Add red, F–G, green, E) to mark spectrosomes and fusomes. Nuclei are marked with DAPI (blue, A–E). p-values were calculated using the two tailed Student's t-test and error bars represent S.E.M., $^{**}p<0.005$. Scale bars, 5 μm.

Biology Open

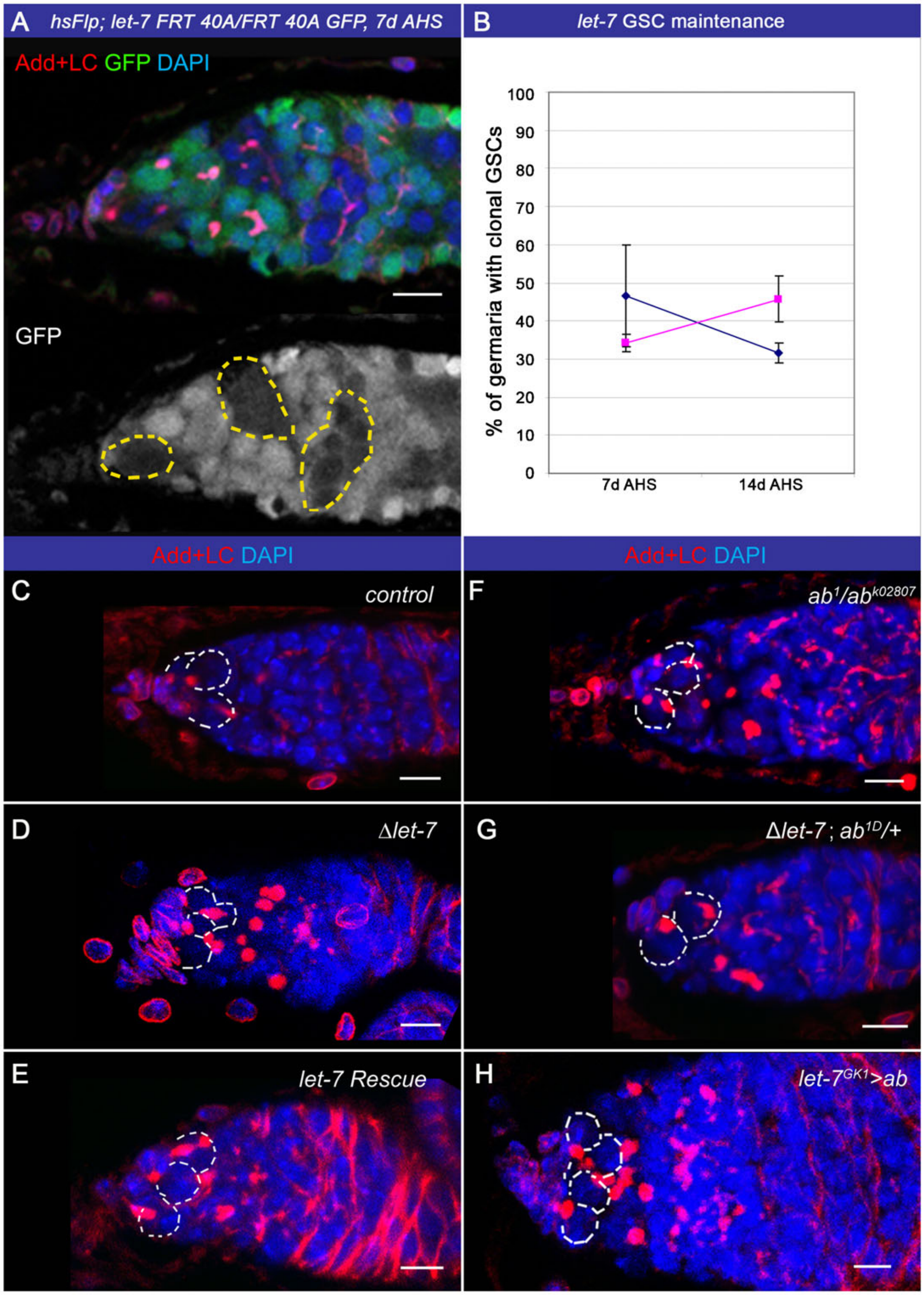

Fig. S5. ***let-7*** **is not required for GSC maintenance, but together with Ab cell non-autonomously influences germline differentiation.** (A,B) The *hsFlp/FRT* system for mitotic recombination was used to induce *let-7* mutant germline cells (*hsFlp/+ ; FRT 40A let-7 miR-125/FRT 40A GFP; let-7-C Δlet-7/+*), clonal cells are marked by the absence of GFP. Parental *FRT 40A* was used as control (*hsFlp/+ ; FRT 40A/FRT 40A GFP*). *let-7* mutation does not affect the maintenance of GSCs. The percentage of the germaria containing at least one *Δlet-7* clonal GSC did not significantly change with the time (34.14±2.24%, n=84 and 45.69±6.10%, n=43 at 7 and 14 days after heat shock, respectively) in comparison to controls (46.67±13.33%, n=81 and 31.71±2.57%, n=62 at 7 and 14 days after heat shock, respectively). (C) Wildtype (*OregonR*) germaria contain on average 4 SpGCs (supplementary material Table S2), (D,E) *let-7* mutants (*let-7-C^{GK1}/let-7-C^{KO1}; let-7-C$^{\Delta let\text{-}7}$/+*) contain a higher number of SpGCs; introducing a *let-7 rescue* construct (*let-7-C/+ ; let-7-C^{GK1}/let-7-C^{KO1}*) reverts the phenotype (supplementary material Table S2). (F) Reducing Ab levels by combining hypomorphic and amorphic alleles leads to an increased number of SpGCs (*ab^{1}/ab^{k0280}*, supplementary material Table S2). (G) The *let-7* mutant phenotype can be partially rescued by reducing *ab* levels [*let-7, miR-125, ab^{1D}/let-7^{KO1}; let-7-C$^{\Delta let\text{-}7}$/+*, compare to panel D, supplementary material Table S2]. (H) Downregulation of Ab using the somatic *let-7^{GK1} Gal4* driver causes an increased number of SpGCs (*let-7^{GK1}/+ ; UASab/+*, supplementary material Table S2). Spectrosomes are marked with Adducin (Add, red, A,C–H), CpCs with LaminC (LC, red, A,C–H), nuclei with DAPI (blue, A,C–H), clones with GFP (green, A). Clonal germline cells are outlined in yellow, GSCs are outlined in white. Scale bars, 5 µm.

Biology Open

Biology Open (2015) 000, 1–16 doi:10.1242/bio.201410553

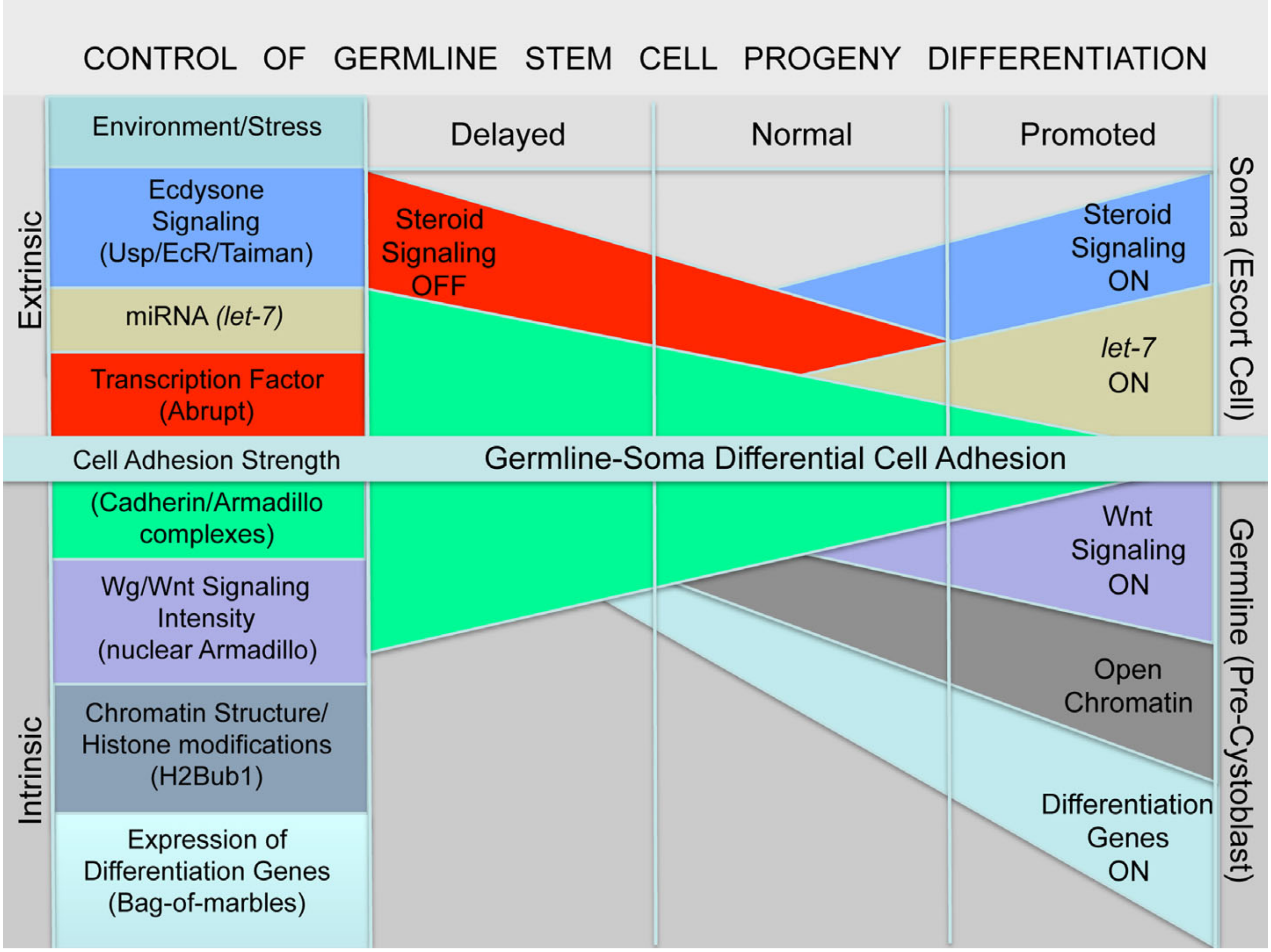

Fig. S6. Scheme of extrinsic and intrinsic signaling controlling early germline differentiation. Control of germline differentiation is highly dependent on precise levels of multiple proteins involved in different signaling pathways that act in the soma and the germline. Ecdysone signaling, miRNA *let-7* and Ab act in the ECs to regulate the adhesion strength between the soma and germline. This, via amounts of Cad/Arm complexes modulates Wg signaling activity in the germline. The Wg pathway establishes specific chromatin status permissive for the differentiation factor Bam expression, leading to GSC progeny differentiation.

Biology Open

Supplementary Table S1. Histone modification (H2Bub1) and ecdysone and Wg signaling defects influence germline differentiation

Genotype, conditions	Spectrosome-containing GCs Ave ± S.E.M.	Fusome-containing cysts Ave ± S.E.M.	Ratio fusome-containing cysts / spectrosome-containing GCs Ave ± S.E.M.	# of analyzed germaria
hsEcR.B1/+,<br>no hs	3.82±0.48	4.45±0.21	1.17±0.05	11
hsEcR.B1/+,<br>2 x 1h hs at 37°C	7.30±0.68<br>[a](p=4.63x10^{-4})***	1.60±0.31<br>[a](p=2.18x10^{-7})***	[b]0.22±0.04<br>[a](p=2.98x10^{-11})***	10
hsbam/+,<br>no hs	4.73±0.33	4.82±0.38	1.02±0.08	11
hsbam/+,<br>2 x 1h hs at 37°C	0.64±0.20<br>[a](p=1.41x10^{-9})***	6.55±0.31<br>[a](p=2.1x10^{-3})**	[b]10.29±0.49<br>[a](p=4.13x10^{-14})***	11
hsEcR.B1/+;<br>*hsbam/+,*<br>no hs	4.82±0.44	4.18±0.26	[b]0.87±0.05	11
hsEcR.B1/+;<br>*hsbam/+,*<br>2 x 1h hs at 37°C	0.80±0.25<br>[a](p=3.05x10^{-7})***<br>[c](p=4.96x10^{-8})***<br>[d](p=0.61)	4.50±0.31<br>[a](p=0.44)<br>[c](p=2.82x10^{-6})***<br>[d](p=1.73x10^{-4})***	[b]5.63±0.38<br>[a](p=7.94x10^{-11})***<br>[c](p=4.12x10^{-11})***<br>[d](p=5.51x10^{-7})***	10
control: nos/+<br>*(NGT40/+;*<br>*nanosGAL4/+),* exp 1	4.90±0.25	6.11±0.21	1.26±0.12	15
nos>Bre1^{RNAi}<br>*(NGT40/+;*<br>*nanosGAL4/Bre^{RNAi})*	4.80±0.30<br>[e](p=8.01x10^{-1})	4.37±0.27<br>[e](p=1.78x10^{-5})***	0.91±0.09<br>[e](p=0.02)*	15
Bre1^{P1549}/+	5.13±0.31	5.27±0.15	1.06±0.05	15
EcR^{Q50st}/+	5.40±0.49	4.33±0.25	0.89±0.09	15
Bre1^{P1549}/EcR^{Q50st}	8.67±0.91<br>[f](p=1.02x10^{-3})**<br>[g](p=3.80x10^{-3})**	3.87±0.31<br>[f](p=3.35x10^{-4})***<br>[g](p=0.25)	0.52±0.07<br>[f](p=1.54x10^{-6})***<br>[g](p=3.71x10^{-3})**	12
nos>fz^{RNAi}<br>*(NGT40/fz^{RNAi};*<br>*nanosGAL4/+)*	5.59±0.41<br>[e](p=0.12)	5.76±0.32<br>[e](p=0.0.37)	1.15±0.13<br>[e](p=0.59)	17
nos>sgg^{RNAi}<br>*(NGT40/sgg^{RNAi};*<br>*nanosGAL4/+)*	4.77±0.47<br>[e](p=0.72)	7.38±0.42<br>[e](p=7.43x10^{-3})*	1.67±0.13<br>[e](p=0.02)*	13
nos>arm<br>*(NGT40/UASarm;*<br>*nanosGAL4/+)*	4.93±0.21<br>[e](p=0.96)	10.25±0.29<br>[e](p=3.37x10^{-13})***	2.17±0.10<br>[e](p=2.98x10^{-7})***	28
nos>arm^{RNAi}<br>*(NGT40/arm^{RNAi};*<br>*nanosGAL4/+)*	6.38±0.46<br>[e](p=0.01)*	[i]n. d.	[i]n. d.	8

Supplementary Table S1. continued

nos>Cad^{RNAi} (NGT40/+; nanosGAL4/Cad^{RNAi})	4.00±0.58 ^{e}(p=0.09)	6.89±0.82 ^{e}(p=0.25)	1.85±0.24 ^{e}(p=0.02)*	9
nos>pan^{RNAi} (NGT40/pan^{RNAi}; nanosGAL4/+)	5.75±0.36 ^{e}(p=0.07)	7.00±0.35 ^{e}(p=0.04)*	1.34±0.10 ^{e}(p=0.61)	16
control: nos/+ (NGT40/+; nanosGAL4/+), exp 2	5.00±0.43	5.85±0.36	1.40±0.13	13
nos>Cad (NGT40/UASp Cad; nanosGAL4/+)	5.93±0.64 ^{h}(p=2.85x10^{-3})**	5.73±0.40 ^{h}(p=0.73)	1.01±0.11 ^{h}(p=1.16x10^{-3})**	15

p-values were calculated using the two tailed Student´s t-test. *p<0.05, **p<0.005,***p<0.0005;
a: compared to the respective genotype without hs;
b: since individual germaria displayed 0 single spectrosome cells, the ratio was calculated by dividing the average number of cysts by the average number of single spectrosome cells;
c: compared to *hsEcR.B1/+;* 2 x 1h hs at 37°C;
d: compared to *hsbam/+;* 2 x 1h hs at 37°C;
e: compared to *control: nos/+,* exp 1;
f: compared to *Bre1^{P1549}/+;*
g: compared to *EcR^{Q50st}/+;*
h: compared to *control: nos/+,* exp 2*;*
i*:* was not determined (n. d.) due to strong phenotypic abnormalities, making it impossible to determine proper regions in the mutant germaria.

Supplementary Table S2. The speed of germline stem cell progeny differentiation depends on the levels of *let-7*, its target Abrupt, and the cell adhesion proteins, DE-Cad and Arm

Genotype	Spectrosome containing GCs Ave ± S.E.M.	Ratio fusome-containing cysts / spectrosome-containing GCs Ave ± S.E.M.	# of analyzed germaria
control: $ab^{1}/+$	4.23±0.32	1.04±0.10	13
ab^{1}/ab^{k02807}	6.00±0.63 $^{a}(p=1.21 \times 10^{-2})^{*}$	0.65±0.08 $^{a}(p=1.58 \times 10^{-2})^{*}$	8
ab^{1}/ab^{1D}	5.50±0.52 $^{a}(p=5.91 \times 10^{-2})$	0.78±0.12 $^{a}(p=1.35 \times 10^{-1})$	16
control: $ab^{RNAi}/+$, 7d at 29°C	5.33±0.40	0.85±0.11	12
$ptc^{ts}>ab^{RNAi}$ ($ptcGal4/ab^{RNAi}$; $tubGal80^{ts}/+$), 7d at 29°C	6.90±0.55 $^{b}(p=2.36 \times 10^{-4})^{***}$	0.71±0.07 $^{b}(p=2.09 \times 10^{-2})^{*}$	10
$bab1^{ts}>ab^{RNAi}$ ($tubGal80^{ts}/ab^{RNAi}$; $bab1Gal4/+$), 7d at 29°C	5.44±0.38 $^{b}(p=2.46 \times 10^{-2})^{*}$	0.79±0.12 $^{b}(p=1.43 \times 10^{-1})$	9
$let\text{-}7\text{-}C^{GK1}/+$, exp 1, 7d at 29°C	5.38±0.30	1.21±0.07	16
$let\text{-}7\text{-}C^{GK1\ ts}>ab$ ($let\text{-}7\text{-}C^{GK1}/+$; $tubGal80^{ts}/UASab$), 7d at 29°C	6.78±0.43 $^{c}(p=1.40 \times 10^{-2})^{*}$	0.75±0.07 $^{c}(p=7.83 \times 10^{-5})^{***}$	18
$ptc^{ts}>ab$ ($ptcGal4/+$; $tubGal80^{ts}/UASab$), 7d at 29°C	7.67±1.21 $^{c}(p=2.87 \times 10^{-2})^{*}$	0.79±0.18 $^{c}(p=1.61 \times 10^{-2})^{*}$	9
control: w^{1118-}	4.28±0.25	1.31±0.10	29
$let\text{-}7\text{-}C^{GK1}/+$, exp 2	5.61±0.23 $^{d}(p=2.49 \times 10^{-4})^{***}$	1.05±0.07 $^{d}(p=3.64 \times 10^{-2})^{*}$	28
$let\text{-}7^{K01}/+$	5.14±0.22 $^{d}(p=1.57 \times 10^{-2})^{*}$	0.93±0.06 $^{d}(p=4.59 \times 10^{-3})^{**}$	22
Δ *let-7* ($let\text{-}7\text{-}C^{GK1}/let\text{-}7\text{-}C^{KO1}$; $let\text{-}7\text{-}C^{\Delta let\text{-}7}/+$), exp 2	6.79±0.25 $^{d}(p= 2.78 \times 10^{-5})^{***}$ $^{e}(p= 1.46 \times 10^{-1})$ $^{f}(p= 2.50 \times 10^{-2})^{*}$	0.55±0.03 $^{d}(p= 6.01 \times 10^{-13})^{***}$ $^{e}(p=2.03 \times 10^{-10})^{***}$ $^{f}(p=8.20 \times 10^{-8})^{***}$	52

Biology Open (2015) 000, 1–16 doi:10.1242/bio.201410553

Supplementary Table S2. continued

let-7 Rescue (let-7-C/+; let-7-C^{GK1}/let-7-C^{KO1})	4.90±0.31<br>[d](p=0.11)<br>[e](p=0.06)<br>[f](p=0.53)<br>[g](p=1.19 x 10^{-2})*	1.43±0.14<br>[d](p=0.47)<br>[e](p=9.09 x 10^{-3})*<br>[f](p=1.38 x 10^{-3})**<br>[g](p=1.10 x 10^{-12})***	20
Δ *let-7 (let-7, miR-125/let-7^{KO1}; let-7-C$^{\Delta let-7}$/+),* exp 3	6.58±0.37	0.67±0.04	26
Δ *let-7, ab* reduced *(let-7, miR-125, ab^{1D}/ let-7^{KO1}; let-7-C$^{\Delta let-7}$/+)*	4.94±0.22<br>[h](p=2.24 x 10^{-4})***	0.94±0.05<br>[h](p=8.77 x 10^{-5})***	31
w^{1118}; hs-Gal4-usp.LBD/+, 4 x 1h hs at 37°C	6.62±0.33	0.62±0.08	13
w^{1118}; hs-Gal4-EcR.LBD/+, 4 x 1h hs at 37°C	6.00±0.27	0.57±0.08	16
shg^{E17B}/+, 4 x 1h hs at 37°C	4.92±0.58	1.14±0.11	13
w^{1118}; hs-Gal4-usp.LBD/ shg^{E17B}, 4 x 1h hs at 37°C	4.57±0.45<br>[i](p=2.17 x 10^{-3})**<br>[k](p=0.47)	1.17±0.20<br>[i](p=8.52 x 10^{-3})*<br>[k](p=0.83)	8
arm^{2}/+, 4 x 1h hs at 37°C	4.31±0.31	1.38±0.15	13
w^{1118}; hs-Gal4-usp.LBD/arm^{2}, 4 x 1h hs at 37°C	4.90±0.35<br>[i](p=2.26 x 10^{-3})**<br>[l](p=0.25)	1.08±0.07<br>[i](p=1.34 x 10^{-4})***<br>[l](p=4.60 x 10^{-2})*	20
w^{1118}; hs-Gal4-EcR.LBD/arm^{2}, 4 x 1h hs at 37°C	5.54±0.40<br>[m](p=0.33)<br>[l](p=2.29 x 10^{-2})*	0.90±0.11<br>[m](p=2.81 x 10^{-2})*<br>[l](p=2.43 x 10^{-2})*	13

p-values were calculated using the two tailed Student´s t-test. *$p<0.05$, **$p<0.005$, ***$p<0.0005$

Compared to:

a: *ab^{1}/+;*
b: *control: ab^{RNAi}/+;*
c: *let-7-C^{GK1}Gal4/+,* exp 1;
d: *w^{1118}*;
e: *let-7-C^{GK1}Gal4/+,* exp 2;
f: *let-7^{K01}/+*;
g: Δ *let-7 (let-7-C^{GK1}Gal4/let-7-C^{K01}; let-7C* Δ *let-7/+),* exp 2;
h: Δ *let-7 (let-7, miR-125/let-7-C^{KO1}; let-7C* Δ *let-7/+),* exp 3;
i: *w^{1118}; hs-Gal4-usp.LBD/+;* 4 x 1h hs at 37°C, exp 1;
k: *shg^{E17B}/+*;
l: *arm^{2}/+;* 4 x 1h hs at 37°C;
m: *w^{1118}; hs-Gal4-EcR.LBD/+;* 4 x 1h hs at 37°C.

Supplementary Table S3. mRNA levels measured by RT-qPCR in *control* and adult-induced *ecdysoneless* mutant

Genotype, conditions	C_T *ab* ± StDev	C_T *RpL32* ± StDev	ΔC_T (ΔC_T *ab*- ΔC_T *RpL32*) [a]	$\Delta\Delta C_T$ ± StDev (ΔC_T- $\Delta C_T^{control}$) [b]	Fold Difference [c]
OregonR germaria 4d at 18°C (control)	31.20±0.13	15.62±0.05	15.57±0.14	0.00±0.14	1.00 (0.91-1.09)
OregonR germaria 4d at 29°C	29.87±0.01	15.62±0.07	14.25±0.07	-1.33±0.07	2.51 (2.39-2.63) [d]($p=4.45 \times 10^{-3}$)**
ecd^{1ts} germaria 4d at 18°C	29.63±0.30	15.52±0.19	14.11±0.36	-1.46±0.36	2.76 (2.16-3.53)
ecd^{1ts} germaria 4d at 29°C	28.03±0.17	15.84±0.08	12.19±0.18	-3.38±0.18	10.44 (9.19-11.86) [d]($p=1.57 \times 10^{-4}$)* [e]($p=6.55 \times 10^{-4}$)***
**Genotype, conditions**	**C_T *arm* ± StDev**	**C_T *RpL32* ± StDev**	**ΔC_T (ΔC_T *arm*- ΔC_T *RpL32*) [a]**	**$\Delta\Delta C_T$ ± StDev (ΔC_T- $\Delta C_T^{control}$) [b]**	**Fold Difference [c]**
OregonR germaria 4d at 29°C	18.78±0.08	13.55±0.36	5.23±0.37	0.00±0.37	1.00 (0.77-1.29)
ecd^{1ts} germaria 4d at 29°C	19.05±0.02	14.78±0.09	4.27±0.10	-0.96±0.10	1.95 (1.82-2.08) [d]($p=4.23 \times 10^{-5}$)
**Genotype, conditions**	**C_T *esg* ± StDev**	**C_T *RpL32* ± StDev**	**ΔC_T (ΔC_T *esg*- ΔC_T *RpL32*) [a]**	**$\Delta\Delta C_T$ ± StDev (ΔC_T- $\Delta C_T^{control}$) [b]**	**Fold Difference [c]**
OregonR germaria 4d at 18°C	32.83±0.50	10.15±0.32	22.67±0.59	0.00±0.59	1.00 (0.66-1.51)
OregonR germaria 4d at 29°C	32.48±0.24	10.28±0.07	22.20±0.25	-0.47±0.25	1.39 (1.17-1.64) [d]($p=0.23$)
ecd^{1ts} germaria 4d at 18°C	31.26±0.15	10.53±0.13	20.72±0.20	0.00±0.20	1.00 (0.87-1.14)
ecd^{1ts} germaria 4d at 29°C	27.44±0.03	9.16±0.03	18.28±0.04	-2.44±0.04	5.43 (5.28-5.59) [d]($p=9.24 \times 10^{-6}$)***

Biology Open

Biology Open (2015) 000, 1–16 doi:10.1242/bio.201410553

Supplementary Table S3. continued

**Genotype, conditions**	**C_T *upd* ± StDev**	**C_T *RpL32* ± StDev**	**ΔC_T (ΔC_T *upd*- ΔC_T *RpL32*) [a]**	**$\Delta\Delta C_T$ ± StDev (ΔC_T-$\Delta C_T^{control}$) [b]**	**Fold Difference [c]**
OregonR germaria 4d at 18°C	23.46±0.01	10.15±0.32[f]	13.31±0.32	0.00±0.32	1.00 (0.80-1.24)
OregonR germaria 4d at 29°C	23.88±0.01	10.28±0.07[f]	13.60±0.07	0.30±0.07	0.81 (0.78-0.86) [d](p=0.42)
ecd[1ts] germaria 4d at 18°C	24.31±0.08	10.53±0.13[f]	13.78±0.16	0.00±0.16	1.00 (0.90-1.11)
ecd[1ts] germaria 4d at 29°C	22.37±0.06	9.16±0.03[f]	13.22±0.06	-0.56±0.06	1.48 (1.41-1.54) [d](p=0.22)
**Genotype, conditions**	**C_T *Imp* ± StDev**	**C_T *RpL32* ± StDev**	**ΔC_T (ΔC_T *Imp*- ΔC_T *RpL32*) [a]**	**$\Delta\Delta C_T$ ± StDev (ΔC_T-$\Delta C_T^{control}$) [b]**	**Fold Difference [c]**
OregonR germaria 4d at 18°C	17.95±0.50	10.15±0.32[f]	7.80±0.59	0.00±0.59	1.00 (0.67-1.50)
OregonR germaria 4d at 29°C	17.33±0.01	10.28±0.07[f]	7.06±0.07	-0.74±0.07	1.68 (1.60-1.76) [d](p=0.14)
ecd[1ts] germaria 4d at 18°C	22.48±0.01	10.53±0.13[f]	11.95±0.13	0.00±0.13	1.00 (0.91-1.10)
ecd[1ts] germaria 4d at 29°C	22.14±0.03	9.16±0.03[f]	12.99±0.04	1.04±0.04	0.49 (0.47-0.50) [d]($p=1.69 \times 10^{-5}$)

a: ΔC_T was determined by subtracting the average *RpL32* C_T value from the average experimental C_T value. The standard deviation of the difference is calculated from the standard deviation of the control and experimental values using the formula $s = \sqrt{(s_1^2 + s_2^2)}$, where s = standard deviation.

b: $\Delta\Delta C_T$ is calculated by subtracting the ΔC_T calibrator value (ΔC_T of the respective *OregonR*). The standard deviation is the same as for ΔC_T.

c: The range of *arm, ab, esg, Upd* or *Imp* is calculated by: $2^{-\Delta\Delta CT}$ with $\Delta\Delta C_T$ +s and $\Delta\Delta C_T$ –s, where s is the standard deviation of $\Delta\Delta C_T$ value.

d, e: ΔC_T values of technical replicates are compared to *OregonR* germaria, 4d at 18°C (d) or *ecd[1ts]* germaria, 4d at 18°C (e).

f: *esg*, *upd* and *Imp* levels levels were measured in the same sample, thus, the same endogenous RpL32 control was used. p-values were calculated using the two tailed Student´s t-test. *p<0.05, **p<0.005, ***p<0.0005

Biology Open (2015) 000, 1–16 doi:10.1242/bio.201410553

Supplementary Table S4. ***let-7*** **levels vary upon different environmental conditions**

Genotype, conditions, age		C_T *let-7* ± StDev	C_T *S2* ± StDev	ΔC_T (ΔC_T *let-7* - ΔC_T *S2*)[a]	$\Delta\Delta C_T$ ± StDev (ΔC_T-ΔC_T ^{control})[b]	Ave $\Delta\Delta C_T$ ± StDev	Fold Difference[c]
control: *OregonR*, Rich food, 2 days	Exp1	25.33±0.30	6.14±0.24	19.19±0.39	0.00±0.39	0.00±0.32	1.00 (0.80-1.25)
	Exp2	26.35±0.29	8.29±0.37	18.06±0.47	0.00±0.47		
	Exp3	28.80±0.34	6.61±0.09	22.19±0.35	0.00±0.35		
	Exp4	27.15±0.39 x10^{-4}	6.89±0.09	20.26±0.09	0.00±0.19		
OregonR, Poor food, 2 days	Exp1	27.06±0.41	7.40±0.29	20.20±0.50	1.01±0.50	-0.40±0.30	1.32 (1.07-1.63)
	Exp2	26.32±0.28	7.96±0.05	18.36±0.29	0.30±0.29		
	Exp3	26.60±0.08	6.28±0.30	20.32±0.31	-1.87±0.31		
	Exp4	25.89±0.09	6.68±0.08	19.22±0.12	-1.04±0.12		
OregonR, 21 days	Exp1	24.32±0.06	6.31±3.46 x10^{-3}	18.00±0.06	-1.19±0.06	-1.09±0.07	2.12 (2.02-2.22) (p=0.01)*
	Exp2	24.96±0.06	7.89±0.04	17.08±0.08	-0.98±0.08		

Genotype, conditions, age	C_T *let-7* ± StDev	C_T *RpL32* ± StDev	ΔC_T (ΔC_T *let-7* - ΔC_T *RpL32*)[a]	$\Delta\Delta C_T$ ± StDev (ΔC_T-ΔC_T ^{control})[d]	Fold Difference[c]
control: *OregonR*, 18°C, 4d	24.33±0.07	16.45±0.25	7.88±0.26	0.00±0.26	1.00 (0.84-1.20)
OregonR, 29°C, 4d	23.64±0.08	17.21±0.50	6.44±0.50	-1.44±0.50	2.72 (1.92-3.85) (p=5.22x10^{-4})***

p-values were calculated using the two tailed Student´s t-test. *p<0.05, **p<0.005, ***p<0.0005;

a: ΔC_T was determined by subtracting the average control C_T value from the average *let-7* C_T value. The standard deviation of the difference is calculated from the standard deviation of the *let-7* and control values using the formula $s = \sqrt{(s_1^2 + s_2^2)}$, where s = standard deviation.

b: $\Delta\Delta C_T$ is calculated by subtracting the ΔC_T calibrator value (ΔC_T of the control: flies 2 days kept on rich food). The standard deviation is the same as for ΔC_T.

c: the range of *let-7* is calculated by: $2^{-\Delta\Delta CT}$ with $\Delta\Delta C_T$+s and $\Delta\Delta C_T$–s, where s is the standard deviation of $\Delta\Delta C_T$ value.

d: $\Delta\Delta C_T$ is calculated by subtracting the ΔC_T calibrator value (ΔC_T of the control: flies kept on 18°C). The standard deviation is the same as for ΔC_T. The fold difference of the experimental values was compared to the respective control.

Biology Open (2015) 000, 1–16 doi:10.1242/bio.201410553

Supplementary Table S5. The efficiency of downregulation of Wg signaling components in the germline by used RNAi mutants

**Genotype**	**C_T *sgg* ± StDev**	**C_T *RpL32* ± StDev**	**ΔC_T (ΔC_T *sgg*- ΔC_T *RpL32*)[a]**	**$\Delta\Delta C_T$ ± StDev (ΔC_T- $\Delta C_T^{control}$)[b]**	**Fold Difference[c]**
control: w[1118]	19.35±0.08	15.89±0.04	3.45±0.09	0.00±0.09	1.00 (0.94-1.06)
nos>sgg[RNAi] (NGT40/sgg[RNAi]; nanosGAL4/+)	19.95±0.05	15.31±0.08	4.64±0.10	1.19±0.10	0.44 (0.41-0.47)
**Genotype**	**C_T *pan* ± StDev**	**C_T *RpL32* ± StDev**	**ΔC_T (ΔC_T *pan*- ΔC_T *RpL32*)[a]**	**$\Delta\Delta C_T$ ± StDev (ΔC_T- $\Delta C_T^{control}$)[b]**	**Fold Difference[c]**
control: w[1118]	19.85±0.13	15.89±0.04[d]	3.96±0.14	0.00±0.14	1.00 (0.91-1.1)
nos>pan[RNAi] (NGT40/pan[RNAi]; nanosGAL4/+)	23.74±0.01	14.75±0.00	8.99±0.01	5.04±0.01	0.03 (0.03-0. 31)
**Genotype**	**C_T *fz* ± StDev**	**C_T *RpL32* ± StDev**	**ΔC_T (ΔC_T *fz*- ΔC_T *RpL32*)[a]**	**$\Delta\Delta C_T$ ± StDev (ΔC_T- $\Delta C_T^{control}$)[b]**	**Fold Difference[c]**
control: w[1118]	22.33±0.04	15.89±0.04[d]	6.44±0.06	0.00±0.06	1.00 (0.96-1.04)
nos>fz[RNAi] (NGT40/fz[RNAi]; nanosGAL4/+)	24.49±0.09	15.09±0.01	9.40±0.09	2.96±0.09	0.13 (0.12-0.14)

a: ΔC_T was determined by subtracting the average *RpL32* C_T value from the average experimental C_T value. The standard deviation of the difference is calculated from the standard deviation of the control and experimental values using the formula $s = \sqrt{(s_1^2 + s_2^2)}$, where s = standard deviation.

b: $\Delta\Delta C_T$ is calculated by subtracting the ΔC_T calibrator value (ΔC_T of the respective w^{1118}). The standard deviation is the same as for ΔC_T.

c: The range of *sgg, pan* and *fz* is calculated by: $2^{-\Delta\Delta CT}$ with $\Delta\Delta C_T$+s and $\Delta\Delta C_T$–s, where s is the standard deviation of $\Delta\Delta C_T$ value.

d: *sgg, pan* and *fz* mRNA levels were measured in the same sample, thus, the same endogenous *RpL32* control was used.

Biology Open (2015) 000, 1–16 doi:10.1242/bio.201410553

Supplementary Table S6. Germline-specific clones of Bre1 and Wg signaling components result in differentiation defects

Genotype	Germaria containing GFP$^-$ clonal germline cells				
	GSC maintenance		GSC progeny differentiation status		
	with clonal GSCs	without clonal GSCs	with clonal cysts delayed in differentiation	with clonal cysts prematurely differentiated	n
control (hsFlp; FRT 2A parental/ FRT 2A GFP)	79.17%	20.83%	4.16%	0.00%	24
Bre1 (hsFlp; FRT 2A Bre1^{P1549}/ FRT 2A GFP)	35.00%	65%	20.00%	0.00%	20
control (FRT 101 parental/ FRT 101 GFP; hsFlp/+)	71.43%	28.57%	0.00%	0.00%	28
arm^2 (FRT 101 arm^2/ FRT 101 GFP; hsFlp/+)	72.23%	27.77%	5.55%	0.00%	18
arm^3 (FRT 101 arm^3/ FRT 101 GFP; hsFlp/+)	29.17%	70.83%	12.5%	0.00%	24
sgg (FRT 101 sgg^{D127}/ FRT 101 GFP; hsFlp/+)	50.00%	50.00%	3.12%	46.87%	32

Differentiation status of clonal mutant cysts (GFP$^-$) was characterized based on fusome morphology and number of cells in the cyst; cysts were defined as delayed in differentiation if the neighboring non-clonal cysts were of a later differentiation stage; cysts were defined as prematurely differentiated if the neighboring non-clonal cysts were of an earlier differentiation stage. In order to analyze the significance between the frequencies of the differentiation stages of clonal germline cells of different genotypes two-way tables and X^2 test were used. The obtained X^2 equals to 68.49, which is greater than the critical value of 18.30, suggesting that there is significant evidence to reject the null hypothesis. This signifies that there indeed is a relationship between the row and the column variables.
